# Inhibition of endotrophin, a cleavage product of collagen VI, confers cisplatin sensitivity to tumours

**DOI:** 10.1002/emmm.201202006

**Published:** 2013-04-30

**Authors:** Jiyoung Park, Thomas S Morley, Philipp E Scherer

**Affiliations:** 1 Departments of Internal Medicine, Touchstone Diabetes Center, The University of Texas Southwestern Medical CenterDallas, TX, USA; 2 Cell Biology, The University of Texas Southwestern Medical CenterDallas, TX, USA; 3 Simmons Cancer Center, The University of Texas Southwestern Medical CenterDallas, TX, USA

**Keywords:** breast cancer, cisplatin, collagen VI, endotrophin, thiazolidinediones

## Abstract

Endotrophin is a cleavage product of collagenVIα3 (COL6A3). Here, we explore the relationship between thiazolidinediones (TZDs), endotrophin and cisplatin resistance in the context of a mammary tumour model. COL6A3 levels are increased in response to cisplatin exposure in tumours. Endotrophin, in turn, causes cisplatin resistance. The effects of endotrophin can be bypassed, either through use of COL6 null (COL6^−/−^) mice or by administering TZDs in wild-type mice (leading to a downregulation of endotrophin). Both approaches sensitize tumours to cisplatin through the suppression of endotrophin-induced epithelial–mesenchymal transition. The beneficial effects of TZDs on cisplatin sensitivity are diminished in COL6^−/−^ mice, whereas endotrophin^+^ tumours are sensitive to the TZD/cisplatin combination. Therefore, the chemosensitization obtained with TZDs is achieved through a downregulation of endotrophin. Treatment with an endotrophin neutralizing antibody in combination with cisplatin completely inhibits tumour growth of tumour allografts. Combined, our data suggest that endotrophin levels are a strong prognostic marker for the effectiveness of the combination therapy of TZDs with cisplatin, and neutralization of endotrophin activity dramatically improves the therapeutic response to combination therapy.

## INTRODUCTION

The platinum-based chemotherapeutic agent cisplatin (*cis*-diammine-dichloro-platinum) has been well established in clinical treatment regimens due to its effectiveness on human tumour cells, such as in the context of ovarian, lung, testicular and breast cancer (Kelland, 2007; Lee et al, 2004; Sirohi et al, 2008). Cisplatin triggers formation of intra-strand and inter-strand DNA-adducts, which leads to cell cycle arrest, followed by apoptosis (Kelland, 2007). However, an inherent or acquired resistance to cisplatin is a major clinical drawback for patients who relapse after initial favourable responses (Galluzzi et al, 2012). Cisplatin resistance is a complex problem which involves multiple pathways including increased drug efflux, evasion of apoptotic pathways, a bypass of the replication checkpoint, increased cell proliferation and increased DNA damage repair (Galluzzi et al, 2012). To overcome the drug resistance against platinum-based chemotherapy, combination therapies with peroxisome proliferator-activated receptor gamma (PPARγ) agonists, the thiazolidinediones (TZDs), have been performed. The basis for this approach is the growth inhibitory effect of these PPARγ agonists on transformed cells through both PPARγ-dependent and -independent pathways (Blanquicett et al, 2008; Mueller et al, 1998; Palakurthi et al, 2001; Satoh et al, 2002). PPARγ is a member of the nuclear hormone receptor superfamily and a key transcription factor for adipogenesis. It is also involved in various physiological processes, such as cell proliferation, angiogenesis, inflammation and lipid partitioning (Tontonoz & Spiegelman, 2008). Combination therapies with TZDs have been shown to display beneficial effects on cancer cell death, while also leading to a reduction of overall systemic toxicity to these chemotherapeutic regimens (Girnun et al, 2007, 2008; Tikoo et al, 2009). However, the detailed molecular basis underlying the beneficial effects of TZDs to platinum treatment has yet to be documented.

In the tumour microenvironment, both stromal and cancer cells contribute to various types of extracellular matrix (ECM) proteins to actively remodel the microenvironment favourably for tumour growth and metastasis. Such ECM proteins include fibronectin, laminin, collagen I (COL1), collagen IV (COL4) and collagen VI (COL6) and these ECM components are markedly modulated in response to chemotherapy (Dangi-Garimella et al, 2011; Sherman-Baust et al, 2003; Su et al, 2007). They have been suggested to cause drug resistance in solid tumours, including small-cell lung cancer, ovarian cancer, pancreatic cancer and breast cancer (Helleman et al, 2008; Rintoul & Sethi, 2001; Sherman-Baust et al, 2003; Shields et al, 2012) through multiple pathways. These include an induction of anti-apoptotic pathways (Sethi et al, 1999), decreased drug transport (Netti et al, 2000) and increased survival signals, such as those mediated through integrin-based pathways (Jean et al, 2011). COL6 is composed of three alpha chains; α1, α2 and α3. Particularly, the α3 chain of COL6 (COL6A3) has been highlighted as a promising candidate triggering drug resistance against platinum-based therapeutics since its levels are vastly increased in the cisplatin-resistant cancer cells *in vitro* (Sherman-Baust et al, 2003; Varma et al, 2005). Nevertheless, the more detailed mechanism underlying how COL6A3 regulates drug-resistance has remained elusive.

Recently, we identified endotrophin, a cleavage product of COL6A3 that is actively involved in mammary tumour progression through enhancing the epithelial–mesenchymal transition (EMT), fibrosis and chemokine activity, thereby recruiting stromal cells to the tumour microenvironment (Park & Scherer, 2012a, b). Notably, all of these activities are associated with acquired drug resistance. In this study, we report increased levels of endotrophin following cisplatin exposure. This causes cisplatin-resistance through enhancing the EMT. Furthermore, endotrophin levels were decreased by combination therapy with TZD, leading to a decrease of EMT, fibrosis and vasculature, thereby enhancing cisplatin sensitivity. In contrast, functional COL6 null mice (COL6^−/−^) that display a reduced EMT over the course of tumour progression, failed to show any added beneficial effects of TZDs to cisplatin. Taken together, these results suggest that the beneficial effects of TZDs on platinum-based chemotherapy are mediated through the inhibition of endotrophin in mammary tumours, and that the neutralization of endotrophin activity is a key determinant to unleash the full beneficial effects of TZDs.

## RESULTS

### Cisplatin augments COL6A3 levels, whereas TZDs cause a reduction

**Figure 1:**
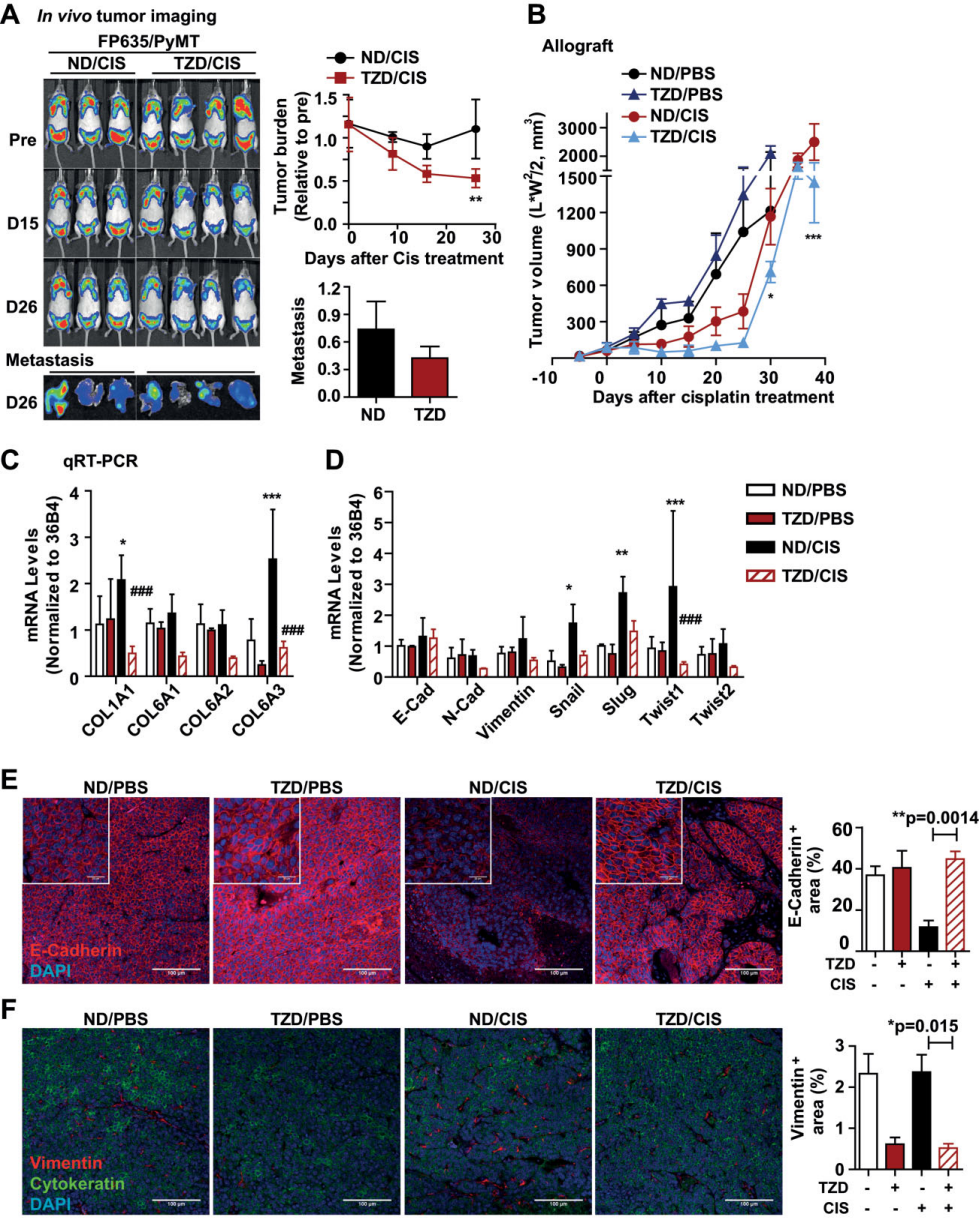
TZD augments cisplatin sensitivity and correlates with the COL6A3 levels **A.** FP635/PyMT mice were given TZD containing chow (supply approx. 20 mg/kg/day, rosiglitazone) or normal-diet (ND) starting at 8-weeks of age, and cisplatin (1 mg/kg) or PBS treatment was initiated at 10 weeks of age (ip., 3 times/week) over the course of tumour progression. Tumour burden was monitored with a fluorescence scanner (IVIS, Caliper life science). Quantified results are represented as mean ± SD (*n* = 8–9/group). *p = 0.04, ND/CIS *versus* TZD/CIS by *unpaired* Student's *t*-test. Metastatic burden was determined by fluorescence signals in lung tissues.**B.** Primary cancer cells isolated from tumours in PyMT mice were implanted into WT mice. TZD were given 5 days prior to cisplatin treatment (1 mg/kg, every 5 days). Tumour volumes were determined by caliper measurement and represented as mean ± SD (*n* = 5–6/group). *p < 0.05 and **p < 0.001, ND/CIS *versus* TZD/CIS by two-way ANOVA.**C,D.** Total RNA was extracted from tumour tissues in each group. mRNA levels for collagens such as COL1A1, COL6A1, -A2 and -A3 (C), and EMT genes such as E-cadherin, N-cadherin, Vimentin, Snail, Slug, Twist1 and Twist2 (D) were determined by qRT-PCR and normalized with 36B4. Quantitative results represent mean ± SD (*n* = 7/group). *p < 0.05, **p < 0.01, ***p < 0.001 ND/PBS *versus* ND/CIS; ^###^*p* < 0.001 ND/CIS *versus* TZD/CIS by two-way ANOVA.**E,F.** EMT indices were determined by immunostaining with E-Cadherin (E) and Vimentin (F). Cytokeratin (epithelial cells) and DAPI (nucleus) were co-stained. Staining positive area was quantified (multiple images, *n* = 5/group). **p = 0.014 (E) and *p = 0.015 (F), ND/CIS *versus* TZD/CIS by unpaired Student's *t*-test. Scale bars: 100 µm.

To assess the beneficial effects of TZD (we are using mostly the TZD rosiglitazone here) on platinum-based chemotherapies in mammary tumour models *in vivo*, we used either a MMTV-PyMT (“PyMT”) mouse model or an allograft of Met-1 cancer cells (originating from MMTV-PyMT mammary tumours) that we transplanted into isogenic wild-type mice. To visualize system-wide tumour burden *in vivo*, we introduced an infrared-fluorescent protein (FP635) overexpressing transgene driven by the MMTV promoter (MMTV-FP635) into PyMT mice (Park & Scherer, 2012b). We monitored tumour regression by utilizing fluorescence scanning over the course of cisplatin treatment (Fig 1A). Consistent with prior reports seen in *in vitro* settings (Girnun et al, 2007), tumour growth was efficiently reduced and pulmonary metastasis were also slightly attenuated in PyMT mice exposed to TZDs (20 mg/kg) in combination with cisplatin (1 mg/kg) compared to those mice given only cisplatin (Fig 1A). Met-1 allografts showed a better response to the combination of TZD with cisplatin than the response seen in PyMT mice (Fig 1B). This may be due to PPARγ-dependent activation of intrinsic oncogenic pathways, such as wnt, or contributions of the tumour stroma responding to a prolonged treatment of TZDs, which may counteract their beneficial effects on cisplatin in the PyMT mice (Saez et al, 2004). In addition, we have previously shown that TZDs are potent inducers of the adipokine adiponectin that we have implicated in enhanced angiogenesis and improved cellular survival (Landskroner-Eiger et al, 2009). Subsequent histological analysis of tumour tissues indicated that cancer cell death was increased about twofold with the TZD combination (Supporting Information Fig S1A). The fact that the metallothionein (MT) levels, a molecular marker for drug resistance (Theocharis et al, 2003), are suppressed by the TZD combination with cisplatin, is well appreciated (Girnun et al, 2007). Consistently, immunostaining for MT in tumour tissues of PyMT mice showed that cisplatin treatment significantly increased the MT levels, and this was suppressed in the presence of TZD (Supporting Information Fig S1B). As such, the PyMT mice serve as a useful model to assess the beneficial effects of TZDs in platinum-based therapeutics *in vivo*.

To see whether COL6 is involved in the beneficial effects of TZDs on platinum-based therapy, we determined the expression levels for COL6 in response to chemotherapy. The mRNA levels of COL6A3 in tumour tissues of PyMT mice were significantly increased in response to cisplatin treatment; this increase was dramatically suppressed by combination with TZDs (Fig 1C). These results indicate that COL6A3 levels may have an impact on the degree of chemo-sensitivity between TZDs and platinum *in vivo*. Nevertheless, whether and how COL6A3 directly contributes to drug responsiveness is not known.

### Cisplatin augments epithelial–mesenchymal transition, whereas TZD attenuates it

The EMT process in tumour tissues is well known to contribute to an acquired drug resistance (Arumugam et al, 2009; Latifi et al, 2011). This suggests a fundamentally reduced sensitivity of mesenchymal-like cells to chemotherapeutic approaches. Targeting the critical factors that contribute to the EMT process, such as Snail, Slug and Twist1, has beneficial effects for cisplatin-based therapies (Haslehurst et al, 2012; Zhu et al, 2012), further generalizing a model that correlates the degree of cisplatin sensitivity with the EMT status of tumour tissues. Moreover, TZDs have been suggested to suppress EMT, resulting in a reduced level of tumour metastasis (Reka et al, 2010). In our mouse models, the mRNA levels for transcription factors associated with EMT, such as Snail, Slug and Twist1, were significantly increased in response to cisplatin exposure. The increases in critical mediators of EMT, especially the increased levels of Twist1, were significantly attenuated by combination treatment with TZD (Fig 1D). This supports the idea that cisplatin induces EMT in cancer cells, and that the beneficial effects of TZDs in the context of cisplatin exposure are partly mediated by suppression of EMT. This is substantiated by immunohistochemistry with critical EMT markers. Immunostaining with antibodies against EMT markers that include either the loss of E-cadherin or an increase in vimentin expression, showed a significant increase of EMT following cisplatin treatment in tumour tissues. The levels of E-Cadherin were sustained the TZD/cisplatin treated group (TZD/CIS) relative to the control group (ND/PBS) (Fig 1E). In parallel, cisplatin-induced increases in vimentin levels were also significantly reduced by combination with TZD (Fig 1F). These observations prompted us to test whether endotrophin plays a critical role in the cisplatin-driven increase of EMT, as endotrophin plays a generalized role in EMT in tumour tissues (Park & Scherer, 2012b). The question therefore is whether we can connect the TZD-mediated decrease in COL6A3 levels (Fig 1C) to the TZD-mediated enhanced cisplatin sensitivity through suppression of the endotrophin-induced EMT.

### Absence of COL6 sensitizes tumours to cisplatin treatment, which is reversed by reconstitution with endotrophin

**Figure 2:**
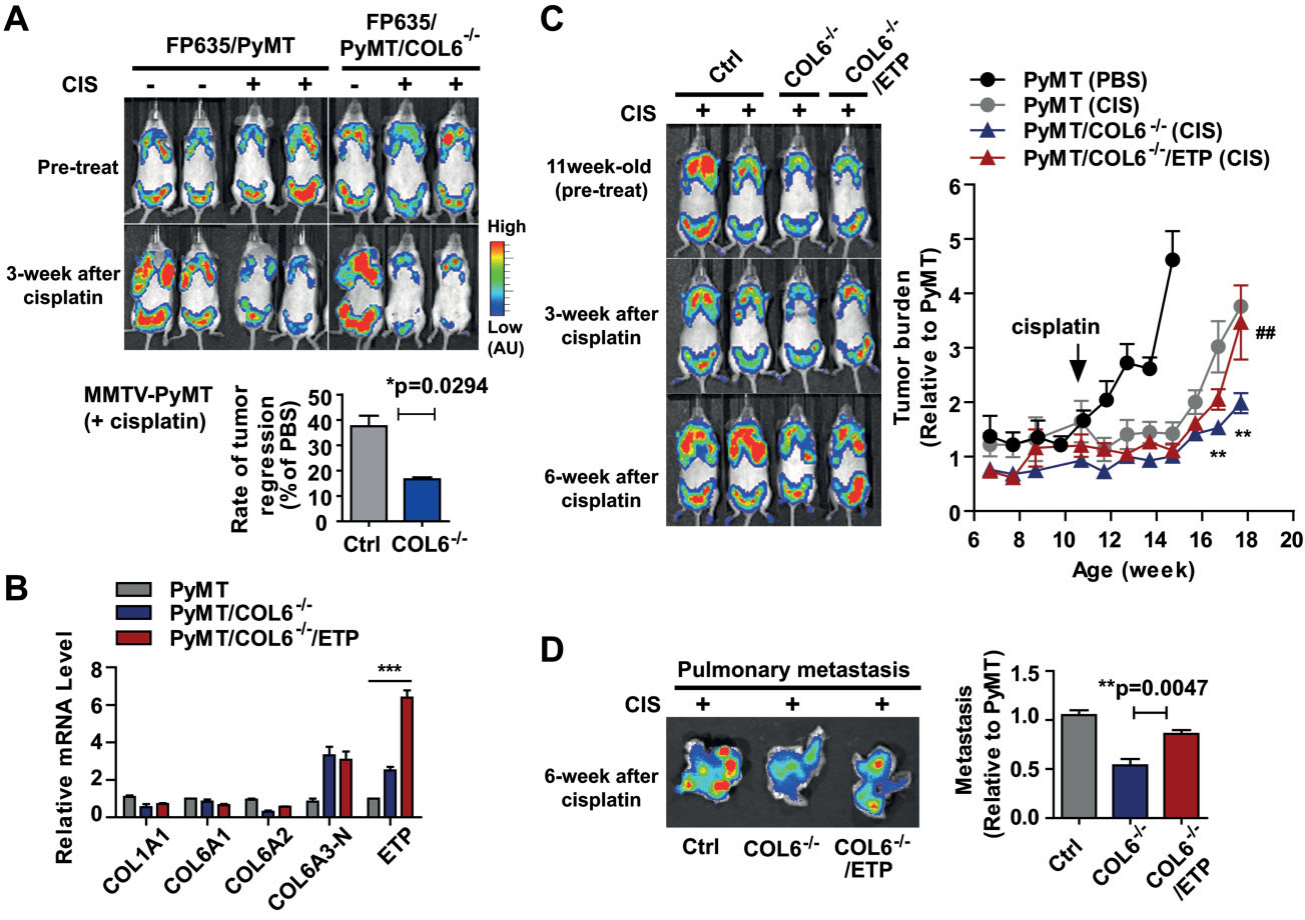
The absence of COL6 in PyMT mice sensitizes tumours to cisplatin treatment **A.** Eleven week old FP635/PyMT and FP635/PyMT/COL6^−/−^ mice were given cisplatin (1 mg/kg, ip., 2 times/week) or PBS over the course of tumour progression. Tumour burden at a whole body level was monitored with a fluorescence scanner (IVIS) once a week. Representative images and quantification showing increased cisplatin sensitivity in PyMT/COL6^−/−^ mice. Tumour burden at the end point was determined and represented as mean ± SD (*n* = 5/group). *p = 0.0294 *versus* FP635/PyMT/CIS by Mann–Whitney *t*-test.**B.** Total RNA was prepared from the tumour tissues from PyMT, PyMT/COL6^−/−^ and PyMT/COL6^−/−^/ETP mice. mRNA levels for the COL1A1, COL6A1, -A2, -A3-N (amino-terminus of COL6A3) and ETP were determined by qRT-PCR. Values were normalized with 36B4 and represented as mean ± SD (*n* = 4/group). Relative values of each gene are represented as fold increase over PyMT. ***p < 0.001 *versus* PyMT by two-way ANOVA.**C,D.** Eleven week old FP635/PyMT/COL6^−/−^ (COL6^−/−^) and FP635/PyMT/COL6^−/−^/Endotrophin (COL6^−/−^/ETP) mice were given cisplatin for 6-weeks compared to PyMT control littermates (Ctrl). Tumour burden was determined by fluorescence signal intensity during the cisplatin treatment (C). Fold increase over PyMT in pretreatment represents mean ± SD (*n* = 5–6/group). **p < 0.01 PyMT (CIS) *versus* PyMT/COL6^−/−^ (CIS); ^##^*p* < 0.01, PyMT/COL6^−/−^ (CIS) *versus* PyMT/COL6^−/−^/ETP (CIS) by two-way ANOVA. Metastatic burden at the end point was determined with lung tissues and represented as mean ± SD (*n* = 5–6/group) (D). **p = 0.0047 COL6^−/−^ *versus* COL6^−/−^/ETP by Mann–Whitney *t*-test.

To assess the roles of COL6 in cisplatin resistance, we performed a loss-of function study by utilizing the COL6^−/−^ mice crossed with PyMT mice (PyMT/COL6^−/−^). PyMT/COL6^−/−^ mice were more sensitive to cisplatin treatment compared to PyMT mice (Supporting Information Fig S2A). Notably, the tumour growth in PyMT/COL6^−/−^ mice is delayed at the early onset of tumour progression (Iyengar et al, 2005). To avoid the effects of COL6^−/−^ on tumour growth *per se*, we compared the chemo-sensitivity with size-adjusted tumours between PyMT/COL6^−/−^ and PyMT mice. Even with size-adjusted tumours, cisplatin-induced tumour regression was clearly enhanced in COL6^−/−^ mice as determined by whole body imaging of tumour burden (Fig 2A). Tumour burden in PyMT/COL6^−/−^ mice was about twofold less than what is seen in PyMT mice 3-weeks post-cisplatin treatment initiation (Fig 2A), arguing that the absence of COL6 in tumour tissues contributes to cisplatin sensitivity. Consistent with these observations, we observed fourfold higher levels of apoptosis in PyMT/COL6^−/−^ mice compared to PyMT mice (Supporting Information Fig S2B), and cisplatin-induced MT levels were also significantly decreased (Supporting Information Fig S2C).

To directly explore the effects of endotrophin in cisplatin responsiveness, we utilized a gain-of function approach by using transgenic mice overexpressing endotrophin, driven by a MMTV-promoter. This limits endotrophin secretion to the local tumour microenvironment (Park & Scherer, 2012b). MMTV-endotrophin transgenic mice were reconstituted into PyMT/COL6^−/−^ mice (*i.e.* PyMT/COL6^−/−^/endotrophin mice). We monitored this strain's cisplatin sensitivity and compared it to either PyMT or PyMT/COL6^−/−^ mice. The mRNA levels for endotrophin in tumour tissues in PyMT/COL6^−/−^/endotrophin mice were about fivefold higher than those of endogenous levels in PyMT mice, whereas no changes were seen at the level of other COL6 chains (−A1 and −A2) or the remaining mature portion of full-length COL6A3 chain (COL6A3-N) (Fig 2B). Importantly, longitudinal measurements of whole body tumour burden in PyMT mice showed that reconstitution of endotrophin into PyMT/COL6^−/−^ mice conferred cisplatin resistance (Fig 2C). Furthermore, the established reduced levels of pulmonary metastasis in PyMT/COL6^−/−^ mice were also reversed by endotrophin reconstitution (Fig 2D). This pinpoints the endotrophin cleavage fragment of COL6A3 as a necessary and sufficient component of the full-length COL6A3 protein to induce cisplatin resistance in otherwise COL6^−/−^ mice. In light of these results, we started to focus our further analysis exclusively on endotrophin, the fragment that we recently identified as a potent tumour-promoting factor (Park & Scherer, 2012b).

### Endotrophin, a cleavage product of COL6A3, confers cisplatin resistance in tumour tissues

**Figure 3:**
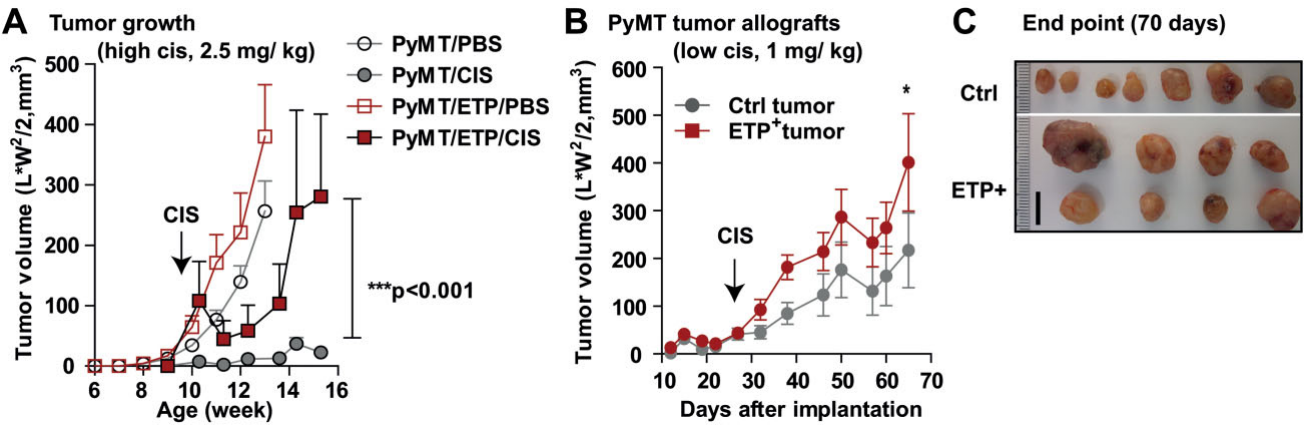
Endotrophin overexpression confers cisplatin resistance in PyMT mice **A.** Ten week old PyMT and PyMT/endotrophin (PyMT/ETP) mice were given high dosage of cisplatin (2.5 mg/kg, ip., 2 times/week). Tumour growth was determined by caliper measurements. Data represent mean ± SD (*n* = 7–10/group). ***p < 0.001, PyMT/CIS *versus* PyMT/ETP/CIS by two-way ANOVA.**B,C.** A piece of tumours taken from PyMT (Ctrl-tumour) and PyMT/endotrophin (ETP^+^-tumour) mice were implanted into isogenic wild-type hosts. Cisplatin (1 mg/kg, ip., 2 times/week) were injected at 3-weeks post-implantation for tumour progression. Tumour volume was determined by caliper measurement. Quantification (B) and representative images (C) showing increased cisplatin resistance in ETP^+^-tumours. Data represent mean ± SD (*n* = 7–8/group). *p < 0.05 *versus* Ctrl-tumours by two-way ANOVA. Representative images were taken at 70-days post-implantation. Scale: 10 mm.

We have previously shown that MMTV-endotrophin mice bred to the PyMT mice (PyMT/endotrophin) develop more aggressive tumours compared to PyMT mice (Park & Scherer, 2012b). Here, we further examined these mice to see whether endotrophin induces cisplatin resistance. PyMT/endotrophin transgenic mice were treated with either PBS or cisplatin and compared to PyMT control littermates. Primary tumour growth of PyMT mice was effectively curbed with a high dosage of cisplatin treatment (2.5 mg/kg, ip. twice a week), whereas PyMT/endotrophin mice were resistant to the effects of cisplatin treatment (Fig 3A). Similarly, allografts of tumour pieces taken from PyMT and PyMT/endotrophin mice transplanted into isogenic wild-type mice showed that endotrophin^+^-tumours were more resistant to a lower dosage of cisplatin treatment (1 mg/kg, ip. twice a week) compared to control (Ctrl)-tumours (Fig 3B and C). These results further corroborate a direct connection of cisplatin-induced high levels of endotrophin expression and chemo-resistance.

### Association of endotrophin with epithelial–mesenchymal transition and chemo-sensitivity

**Figure 4:**
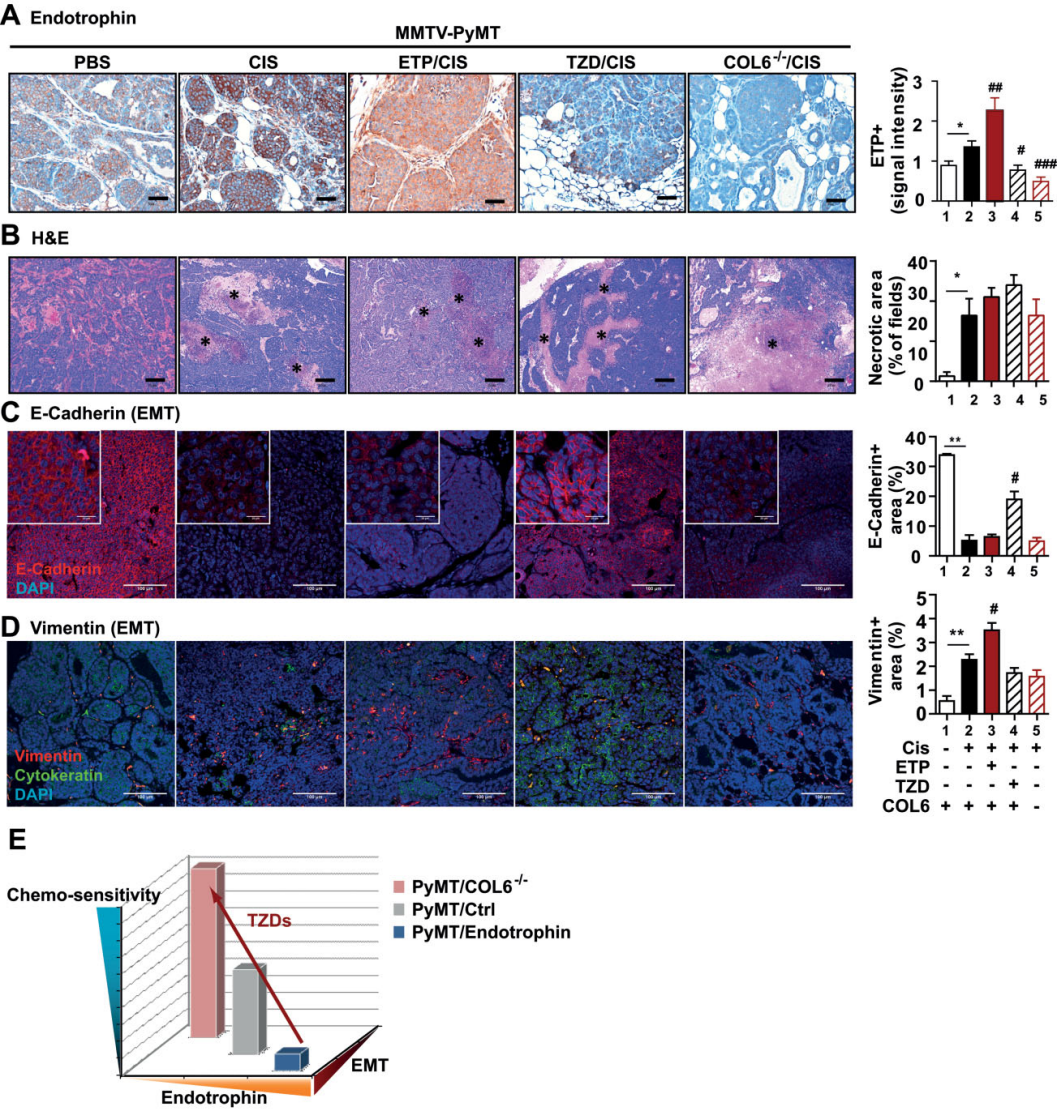
Histological analysis of tumours in PyMT mice with different levels of endotrophin after chemotherapy Endotrophin staining and quantification, showing increased endotrophin levels upon cisplatin treatment which was further augmented in PyMT/endotrophin mice, whereas it was barely detectable in PyMT/TZD and PyMT/COL6^−/−^ mice. **p = 0.0174, ^##^*p* = 0.009, ^#^*p* = 0.024 and ^###^*p* = 0.0004.H&E staining and necrotic lesion area quantification on tumours, showing increased cell death after cisplatin treatment in all groups, and further augmented sensitivity in PyMT/TZD. *p = 0.0463.E-cadherin staining and quantification, showing decreased membrane integrity of epithelial cancer cells after cisplatin treatment in PyMT mice. TZD reverses cisplatin-induced loss of E-cadherin levels. **p = 0.0045 and ^#^*p* = 0.0285.Vimentin staining and quantification, showing increased EMT in PyMT/endotrophin mice, whereas it was decreased in PyMT/TZD and PyMT/COL6^−/−^ mice. **p = 0.0085 and ^#^*p* = 0.0111. Quantified results represent mean ± SD (multiple images from *n* = 5–6/group). Statistics (*PBS *vs.* CIS; ^#^CIS *vs.* ETP/CIS, TZD/CIS, or COL6^−/−^/CIS) were analysed by unpaired Student's *t*-test. Scales: 50 µm (A), 200 µm (B) and 100 µm (C–D).Hypothetical modeling of cisplatin responsiveness in PyMT mice relying on the endotrophin levels and EMT status. Arrow indicates TZD augments chemo-sensitivity through suppression of endotrophin levels and EMT.

Endotrophin has multiple effects on tumour progression through TGF-β-dependent EMT and fibrosis, as well as TGF-β-independent chemokine activities that trigger the recruitment of endothelial cells and macrophages, resulting in enhanced angiogenesis and inflammation (Park & Scherer, 2012b). Having implicated endotrophin as a critical mediator of cisplatin resistance, we wanted to further substantiate the endotrophin-induced EMT as a downstream readout for cisplatin resistance acquisition by utilizing a histological approach. Endotrophin levels in PyMT mice were modulated genetically with MMTV-endotrophin transgenic mice for overexpression and with COL6^−/−^ mice to reduce the levels. Alternatively, endotrophin levels were reduced pharmacologically with TZD treatment. Consistent with the mRNA levels of endotrophin seen in Fig 1C, cisplatin significantly increased the endotrophin protein levels in tumour tissues (Fig 4A). In contrast, endotrophin protein levels were dramatically reduced in tumour tissues of both COL6^−/−^ and TZD treatment groups compared to controls (Fig 4A). Histologically, we observed that necrotic lesion areas were increased by cisplatin treatment in all groups (Fig 4B) regardless of the levels of endotrophin in the PyMT setting. Immunostaining for the loss of E-cadherin showed a significant increase of EMT following cisplatin treatment in all groups except for the group with the combination therapy with TZD (Fig 4C). This suggests that the cisplatin-induced EMT is curbed by TZD treatment. Consistent with this observation, immunostaining for vimentin (a mesenchymal cell marker), reveals significantly increases in response to cisplatin. This increase in vimentin was further augmented by endotrophin, whereas both COL6^−/−^ and TZD groups displayed decreased vimentin levels (Fig 4D). This suggests that the cisplatin-mediated acquisition of mesenchymal cell-like traits during the EMT process is linked to endotrophin levels. This also indicates that the modulation of endotrophin levels by either genetic or pharmacological means is tightly associated with EMT levels in tumour tissues, and this correlative decrease of endotrophin and EMT at least partly accounts for the increased cisplatin sensitivity observed in the COL6^−/−^ or TZDs combination groups (Fig 4E).

### Acquisition of the beneficial effects of TZDs to cisplatin critically depends on the endotrophin levels

**Figure 5:**
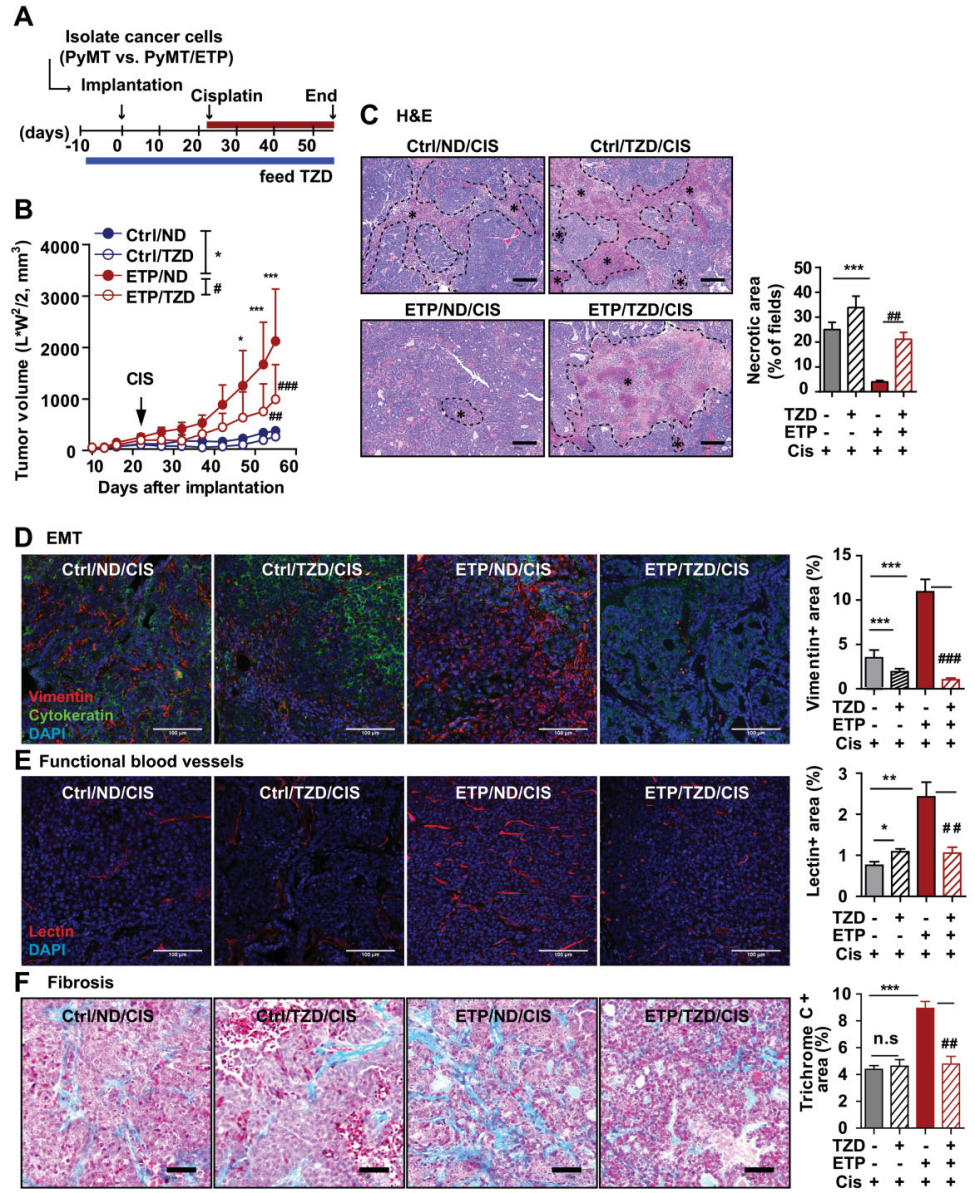
TZD enhances cisplatin sensitivity through suppression of endotrophin-mediated EMT, fibrosis and angiogenesis **A,B.** Schematic diagram for allografts (A), indicating cancer cells were isolated from tumours in PyMT (Ctrl) and PyMT/endotrophin (ETP) mice and implanted into wild-type hosts (0.5 × 10^6^ cells/mouse). Host mice were given TZD (20 mg/kg) or ND diet at 10 days before implantation for tumour progression. Cisplatin (1 mg/kg, ip., every 5 days) was administered at 3-weeks post-implantation. Quantification of tumour volume (B), showing TZD suppressed tumour growth in endotrophin^+^-tumours. Data represent mean ± SD (*n* = 8–9/group). *p = 0.05, **p = 0.01 and ***p = 0.001 Ctrl/ND *versus* ETP/ND; ^##^*p* = 0.01 and ^###^*p* = 0.001 ETP/ND *versus* ETP/TZD by unpaired Student's *t-*test.**C–F.** Histological analysis of tumours in allografts after cisplatin treatment. H&E staining and necrotic area quantification (C), showing significantly increased chemo-sensitivity in endotrophin^+^ tumours upon combination of TZD with cisplatin. Necrotic area (*). ***p < 0.0001 and ^##^*p* = 0.0018. Vimentin staining quantification (D), showing decreased EMT in both Ctrl- and endotrophin^+^-tumours by TZD. ***p < 0.0001 and ^###^*p* < 0.0001. Quantification of perfused lectin staining (E), showing the increased functional blood vessels in endotrophin^+^-tumours was decreased by TZD. *p = 0.015, **p = 0.001 and ^##^*p* = 0.0088. Masson's Trichrome C staining quantification (F), showing increased fibrosis in endotrophin^+^-tumours was decreased by TZD. ***p < 0.0001 and ^##^*p* = 0.0013. Scales: 200 µm (A), 100 µm (D–E) and 50 µm (F). Statistics (*Ctrl/ND/CIS *vs.* Ctrl/TZD/CIS or ETP/ND/CIS; ^#^ETP/ND/CIS *vs.* ETP/TZD/CIS) were analysed by unpaired Student's *t*-test.

We have shown that the beneficial effects of TZDs on the cisplatin therapeutic efficiency are linked to endotrophin down-regulation. Do the TZD effects converge on to the endotrophin-mediated signaling pathways? Both mRNA and protein levels for endotrophin were dramatically reduced with the TZD and cisplatin combination treatment (Figs 1C and 4A, respectively). Therefore, we assessed whether endotrophin overexpression could abolish the beneficial effects of TZD on cisplatin efficacy. Endotrophin^+^-cancer cells originating from PyMT/endotrophin mice were compared to Ctrl-cancer cells from PyMT mice, and were implanted into wild-type mice. TZD was given to wild-type hosts 10 days prior to implantation and cisplatin was injected intraperitoneally every 5 days, starting 3-weeks post-implantation when the tumour volume reached 100 mm^3^ (Fig 5A). Endotrophin^+^-tumours were more resistant to cisplatin treatment compared to Ctrl-tumours (Fig 5B, Ctrl/ND *vs.* ETP/ND), and this increase was markedly attenuated by the combination with TZD (Fig 5B, ETP/ND *vs.* ETP/TZD). This suggests that TZD influences not only the endotrophin expression *per se*, but it may also impact the downstream pathways of endotrophin. However, we cannot rule out that endotrophin-independent pathways are also contributing, or that TZD acts on host endotrophin levels in this transplantation paradigm. Defined necrotic lesion areas, as assessed by H&E stains, were significantly decreased in endotrophin^+^-tumours. This phenomenon was however reversed by combined treatment of cisplatin with TZD (Fig 5C). This suggests that a combination of TZD with cisplatin confers sensitivity to endotrophin^+^-tumours. Accordingly, the significant endotrophin-mediated increase on EMT, angiogenesis and fibrosis seen in endotrophin^+^-tumours was suppressed by the combination of cisplatin with TZD, as judged by immunostaining for vimentin (EMT), lectin perfusion (angiogenesis) and Masson's trichrome C stain (fibrosis), respectively (Fig 5D–F). This suggests that TZD attenuates the downstream signaling pathways induced by endotrophin.

**Figure 6:**
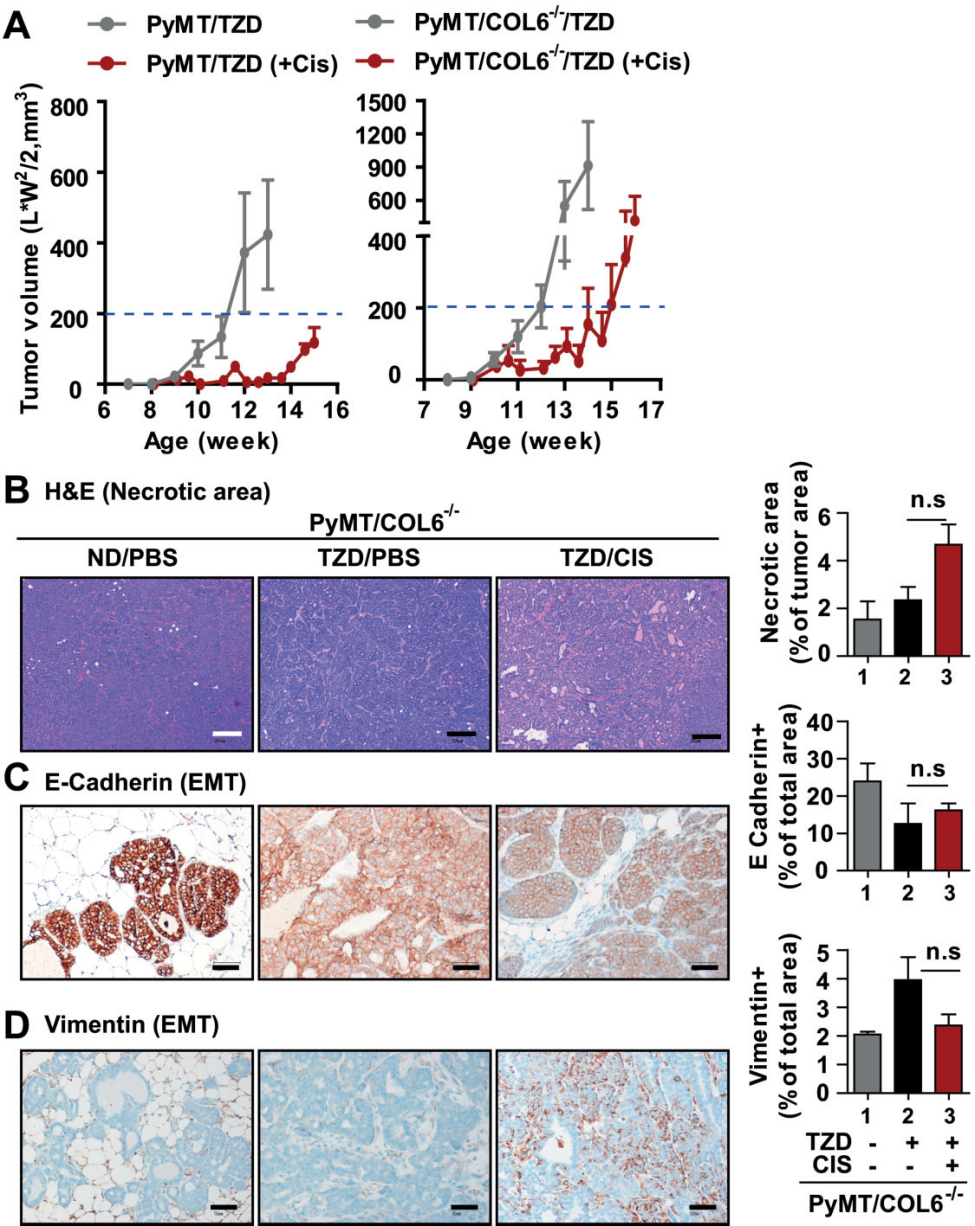
Bypassing endotrophin-downstream pathways with COL6^−/−^ mice abolishes beneficial effects of TZD on cisplatin sensitivity **A.** PyMT/COL6^−/−^ mice were given TZD (20 mg/kg) or ND at 8 weeks of age. Cisplatin (1 mg/kg, ip., 2 times/week) or PBS treatment was initiated in 10 week old mice. Tumour growth was determined by caliper measurements, and PyMT littermates given TZD were represented as a control. Data represent mean ± SD (*n* = 6–8/group).**B–D.** Histological analysis. H&E staining and necrotic area quantification (B), showing no necrotic area in PyMT/COL6^−/−^ mice following TZD combination with cisplatin treatment. EMT was determined by immunostaining for E-Cadherin (C) and Vimentin (D), showing no significant effects in TZD/CIS groups comparable to non-treated groups (PBS). Data represent mean ± SD (multiple images from *n* = 5–6/group). *p* = n.s (no significant) *versus* PyMT/COL6^−/−^/TZD by unpaired Student's *t*-test. Scales: 200 µm.

Given that the endotrophin-mediated downstream signaling is suppressed by TZDs, we predicted that COL6^−/−^ mice would have a remaining, though reduced benefit from a TZD combination with cisplatin. To address this point, we assessed the TZD impact on cisplatin treatment in PyMT/COL6^−/−^ mice and compared them to PyMT mice. Indeed, the beneficial effects of TZD on cisplatin sensitivity were not seen in PyMT/COL6^−/−^ mice, *i.e.* tumour regression was unaffected by TZD combined with cisplatin treatment in PyMT/COL6^−/−^ (Fig 6A). Necrotic lesions were barely detectable in the PyMT/COL6^−/−^/TZD/CIS group relative to PyMT/COL6^−/−^ or PyMT/COL6^−/−^/TZD groups (Fig 6B). As expected, the TZD effects on EMT suppression were not observed in PyMT/COL6^−/−^ mice (Fig 6C–D). These results lend further support to the notion that the beneficial effects of TZDs are critically dependent on the suppression of endotrophin activity. In other words, the endotrophin levels in association with progression towards EMT are critical determinants of the beneficial effects of TZDs on cisplatin treatment.

### The suppression of endotrophin activity can be achieved by either using TZD or anti-endotrophin monoclonal antibodies, both of which sensitize tumours to cisplatin therapeutics

**Figure 7:**
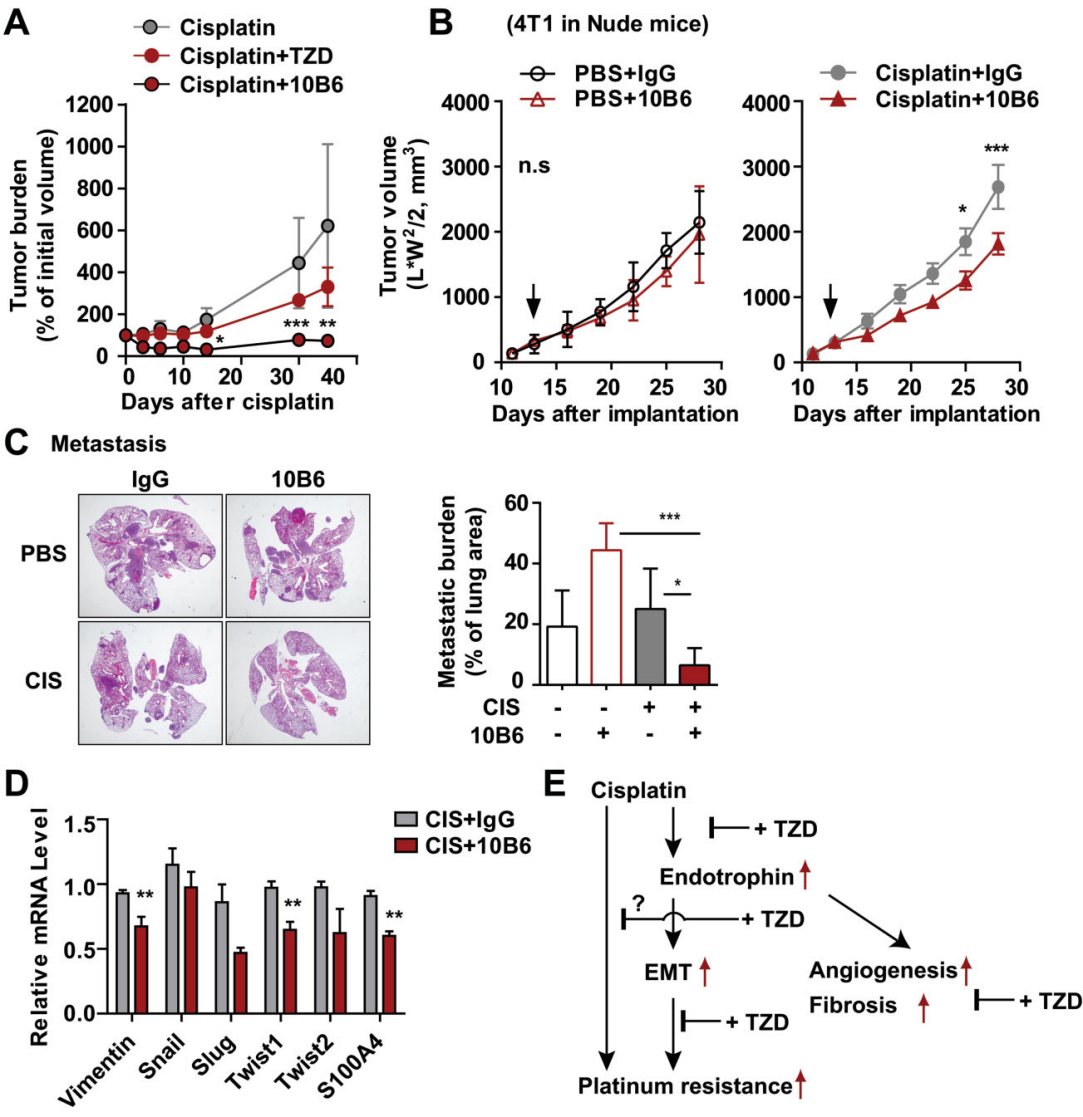
Neutralizing endotrophin activity with monoclonal antibodies sensitizes tumours to cisplatin treatment **A.** Pieces of tumours from PyMT mice were implanted into wild-type hosts. Tumour-bearing mice were given cisplatin (1 mg/kg, ip., every 5 days) or PBS, combined with either TZD (20 mg/kg) or anti-endotrophin monoclonal antibodies (100 µg/mouse, once a week) for tumour progression. Tumour volumes were determined by caliper measurements. Data represent mean ± SD (*n* = 5/group). *p < 0.05, **p < 0.01, ***p < 0.001 *versus* Cisplatin by two-way ANOVA.**B–D.** 4T1 (0.5 × 10^6^ cells/mouse) cells were xenografted in nude mice and monitored tumour growth (B) and metastasis (C). Cisplatin (1 mg/kg, every 5 days, i.p) with either 10B6 or IgG control (100 µg/mouse, once a week, i.p) was given to tumour-bearing mice from 12-days after implantation. Tumour volumes were determined by caliper measurements. Data represent mean ± SD (*n* = 5/group). *p < 0.05, ***p < 0.001 *versus* Cisplatin + IgG by two-way ANOVA. Metastatic burden was determined by measuring metastatic lesion area in lung tissues with H&E stains. Quantified data represent mean ± SD (*n* = 5/group). ***p = 0.0007 and *p = 0.0209 *versus* CIS/10B6 by unpaired Student's *t*-test. mRNA levels for EMT markers such as vimentin, snail, slug, twist1, twist2 and S100S4 were determined by RT-qPCR (D). Values are normalized with 36B4 and represented as mean ± SD (*n* = 5/group). **p = 0.0070, **p = 0.0045, **p = 0.0022 for vimentin, twist1 and S100A4, respectively by unpaired Student's *t*-test.**E.** Summary of the study. Increased endotrophin following cisplatin treatment confers cisplatin resistance, and beneficial effects of TZDs on cisplatin sensitivity are mediated through both a suppression of endotrophin levels and its downstream pathways, including EMT, fibrosis and angiogenesis.

As a last step, we determined therapeutic potential of a previously described endotrophin-neutralizing antibody (clone 10B6) on cisplatin sensitivity. Tumour pieces taken from PyMT mice were implanted into wild-type mice and treated with cisplatin alone or in combination with either TZD or 10B6 once the tumour volume reached 100 mm^3^. Tumour regression was monitored for 2-months post-implantation. We see that both TZD and 10B6 treatment efficiently sensitized the tumours to cisplatin treatment (Fig 7A). We also utilized xenograft models with the mammary carcinoma cell line 4T1, which is highly invasive and rapidly metastasizes throughout the body, resembling human stage IV breast cancer (Pulaski & Ostrand-Rosenberg, 1998). Nude mice were injected with 4T1 cells in mammary adipose tissues. Treatment with cisplatin was initiated when the tumour volume reached at 100 mm^3^, which was combined with either control IgGs or 10B6. Treatment of either 10B6 or cisplatin alone for 28-days barely inhibited primary tumour growth of 4T1, while treatment with cisplatin combined with 10B6 induced a moderate, but significant inhibition in comparison to cisplatin or 10B6 alone (Fig 7B). However, most prominent effects of combination treatment (cisplatin and 10B6) were observed on metastatic growth. The metastatic burden on the lung, as determined by assessing the metastatic lesion areas, was significantly attenuated by combination treatment relative to individual treatments (Fig 7C). Notably, the combination of cisplatin and endotrophin neutralization showed a particularly higher efficacy on metastatic growth than either treatment alone for late stages of 4T1 carcinomas. Furthermore, a subset of genes related to EMT that includes Vimentin, Twist1 and S100A4 levels were also significantly decreased by the combination treatment (Fig 7D). This indicates that inhibitory effects of combination treatment of cisplatin with endotrophin neutralization mediate a suppression of EMT, lack of which results in loss of crucial traits for metastatic growth.

## DISCUSSION

Building on our previous studies, we tested the cellular responses to endotrophin on chemo-responsiveness in mammary tumours treated with cisplatin. We demonstrate that a robust response of cancer cells to cisplatin is highly dependent on the presence of the endotrophin-driven EMT process. Endotrophin overexpression, leading to enhanced EMT, causes cisplatin resistance. In contrast, the suppression of endotrophin levels by either using a COL6^−/−^ mouse model or a TZD combination sensitizes cancer cells to cisplatin treatment. Our data presented here suggests that determining endotrophin levels in association with the EMT status is critical for predicting cisplatin response. Higher levels of endotrophin occur in advanced metastatic breast cancers (Iyengar et al, 2005) and contribute to the poor chemo-response. It also suggests that this subset of tumours is likely to undergo EMT, which plays a major role in tumour progression, metastasis and multi-drug resistance in various epithelial cancer cells (Haslehurst et al, 2012; Latifi et al, 2011; Rosano et al, 2011). Furthermore, we propose that obesity is one of the major risk factors to provide an endotrophin-enriched tumour microenvironment, because it is mainly secreted from adipose tissue and elevated in dysfunctional adipose tissue. Therefore, it will be interesting to see if the endotrophin-mediated EMT we described here is also predictive of a poor chemotherapeutic response in other types of cancers.

Our results are consistent with clinical studies showing that cancer cells with high levels of COL6A3 show a reduced response to platinum-based chemotherapy than tumours with low levels of COL6A3 (Bonaldo et al, 1998; Iyengar et al, 2005; Sherman-Baust et al, 2003; Varma et al, 2005). However, evidence for a direct connection between these two phenomena was lacking. It has been appreciated that there is an enormous degree of ECM remodeling going on in response to chemotherapy, and this in turn has an impact on drug penetration, which critically affects chemo-sensitivity. In addition, increased tissue stiffness seems to confer survival signals to cancer cells through enhanced anchoring of cancers to ECMs. Beyond these purely mechanical roles of ECM remodeling, we found here that endotrophin acts as a signaling molecule leading to an enhanced EMT process, resulting in cisplatin resistance.

The beneficial effects of the combination of TZDs with platinum-based chemotherapy are appreciated. Based on our data, TZD monotherapy fails to have an impact on tumour progression in PyMT mice, and in fact further enhances growth. This is consistent with clinical reports that failed to see an impact on the malignancies of epithelial cancer cells (Burstein et al, 2003; Kulke et al, 2002; Smith et al, 2004). However, TZDs in combination with cisplatin are highly beneficial. How do TZDs enhance cisplatin effectiveness? Here, we show that the beneficial effects of TZDs on cisplatin therapies are due to marked reduction of the endotrophin levels. This attenuates the downstream consequences of endotrophin signaling, including a suppression of EMT, fibrosis and angiogenesis, thereby leading to an increase of chemo-sensitivity (Fig 7E). Therefore, a treatment criterion for a TZD/cisplatin combination therapy would be high levels of endotrophin in association with EMT, due to the fact that the beneficial effects of TZDs are acquired through a direct suppression of endotrophin-induced EMT (Fig 4E). Along those lines, we show that neutralizing endotrophin activity through the use of neutralizing monoclonal antibodies during cisplatin treatment effectively inhibits the tumour growth and metastasis.

**The paper explained**

PROBLEM:

The therapeutic benefit of cisplatin in human cancer treatments is often limited due to resistance. TZDs (peroxisome proliferator activated receptor γ agonists) show beneficial effects in the context of cisplatin-based chemotherapy. Our previous work indicates that collagenVIα3 (COL6A3) plays an important role in cisplatin resistance. However, the detailed molecular mechanisms underlying the correlations between COL6A3 and cisplatin resistance remained elusive. The goal of this study was to elucidate the roles of endotrophin, a cleavage product of COL6A3, in cisplatin resistance and elaborate further to see if endotrophin modulates the beneficial effects of TZDs in cisplatin therapeutics in breast cancer.

RESULTS:

Gain- and loss-of function studies were conducted with MMTV-endotrophin transgenic and COL6 null animals (COL6^−/−^) in the background of MMTV-PyMT mice, respectively. We demonstrate that endotrophin, which is mainly secreted from stromal adipocytes in the tumour microenvironment, confers a high degree of cisplatin resistance by enhancing epithelial–mesenchymal transition, fibrosis and angiogenesis. Furthermore, the powerful beneficial effects of TZDs on cisplatin sensitivity are mainly due to a marked inhibition of endotrophin-mediated activities. This suggests that TZDs directly mediate enhanced cisplatin chemosensitivity through a downregulation of endotrophin. Treatment with an endotrophin neutralizing monoclonal antibody in combination with cisplatin very effectively inhibits tumour growth of allografts of MMTV-PyMT tumours.

IMPACT:

It is well appreciated that chemo-responsiveness is changed over the course of tumour progression, and it varies greatly between different tumour types; identifying the critical players mediating this chemo-resistance is important to devise better therapeutic strategies. Our results have clinical implications, as endotrophin is increased in tumours upon chemotherapy, and the associated EMT is a predictor of chemo-resistance. Therefore, endotrophin levels can be a strong prognostic marker with respect to the tumour response to combination therapy of TZDs with cisplatin, and the neutralization of endotrophin further improves the therapeutic response to combination therapy.

In summary, we have employed a rodent model for a chemotherapeutic tumour response, and demonstrated that the endotrophin-mediated induction of the EMT results in chemo-resistance. Furthermore, we highlighted that the beneficial effects of TZDs on cisplatin-based therapies are mediated through the suppression of this pathway. Bypassing the endotrophin-induced EMT in the context of COL6^−/−^ mice diminished the TZDs-mediated beneficial effects on cisplatin therapeutics and resulted in a poor response. These results provide a direct explanation for previous correlations reported in the context of poor responses to platinum-based chemotherapy in tumours expressing high levels of COL6. This also suggests that endotrophin levels as a promising predictive marker to decide if a TZD combination should be initiated along with a platinum-based therapeutic approach.

## MATERIALS AND METHODS

### Animal experiments

All animal experiments were approved by the Institutional Animal Care and Research Advisory Committee at the University of Texas Southwestern Medical Center. MMTV-PyMT mice (Guy et al, 1992) were used as a mouse mammary tumour model. COL6 knockout (COL6^−/−^) mice were generated as previously described by Bonaldo et al (1998). MMTV-endotrophin transgenic mice and MMTV-FP635 (Infrared fluorescent protein FP635) transgenic mice were generated as previously described in our study (Park & Scherer, 2012a). All experiments were conducted using littermate-controlled female mice. All animals used in this study are in a pure FVB background.

### Reagents

Cisplatin (Sigma, 479306) was diluted to 1 mg/ml in PBS and was sonicated briefly before injection. The PPARγ agonist rosiglitazone (Avandia, GlaxoSmithKline) was given by diet inclusion at a dose of 20 mg/kg/day BW. Anti-mouse endotrophin monoclonal antibodies (10B6, 100 µg/mouse) were administered by intraperitoneal injection.

### Histological analysis

Formalin-fixed paraffin-embedded tissue sections were used for immunostaining. Deparaffinized tissue slides were stained with rabbit anti-mouse endotrophin, MT (Abcam, Ab12228), E-cadherin (Cell signaling, 24E10), Vimentin (Cell signaling, D21H3) and cytokeratin (Cell Signaling, #4545). For immunofluorescence, fluorescence labeled secondary antibodies were used and counterstained with DAPI. Images were acquired using the Leica confocal microscope and analysed with ImageJ software. For immunohistochemistry, the reaction was visualized by the DAB Chromogen-A system (Dako Cytomation) and counterstained with haematoxylin. Images were acquired using the Nikon Cool Scope. TUNEL assay was according to the manufacturer's protocol (Trevigen, Inc). To assess functional blood vessels formation in tumour tissues, mice were injected with biotinylated tomato-lectin (100 µg, i.v) (Vector laboratories, CA) and perfused lectin was visualized by a Cy3-labeled streptoavidin. H&E staining and Masson's Trichrome C staining were performed by Dr. John Shelton at the University of Texas Southwestern Medical Center. Histological analysis was performed with pathologists in the UTSW pathology core facility.

### Quantitative RT-PCR

Total RNA was isolated following tissue homogenization in Trizol (Invitrogen, Carlsbad, CA) using a TissueLyser (Qiagen, Valencia, CA) and isolated using the RNeasy kit (Qiagen). Total RNA (1 µg) was reverse transcribed with SuperScript III reverse transcriptase (Invitrogen). Quantitative real-time PCR (qRT-PCR) was performed in the Roche Lightcycler 480. For all qRT-PCR experiments, the results were calculated using the ΔΔ*C*_t_ method using 36B4 to normalize. Primers for COL1A1, COL6A1, COL6A2 and COL6A3 were followed in previous report (Khan et al, 2009). Other primer sequences used in this study are listed in Supporting Information Table S1.

### Primary culture of mammary cancer cells and implantation

Mammary epithelial cancer cells were isolated as described in previous report (Park et al, 2010). One day after cell culture, same amount of cancer cells were counted and implanted into inguinal fat-pad of 8-to 10-week-old indicated recipient mice by intraductal injection. Tumour growth was determined from 10 days after implantation and twice a week over the course of tumour progression.

### Analysis of tumour progression

Tumour onset was monitored twice weekly by palpation. Tumour sizes were measured with a digital caliper twice weekly and the volumes were calculated as (length × width^2^)/2. Inguinal tumours was weighted to determine tumour burden. Animals were sacrificed when the tumour burden visibly affected the host or when the tumours reached the IACUC predetermined limit of 20 mm along one axis.

### Tumour imaging

Infrared fluorescence expressing MMTV-PyMT mice (FP635/PyMT) were imaged by IVIS scanner (Caliper lifesciences) and signal intensity was analysed with Living image v.3.2 (Caliper lifesciences).

### Statistical analyses

All data represent mean ± SD. Data were analysed by two-way ANOVA followed by Newman–Keuls multiple comparison test or by Student's *t*-test and Mann*–*Whitney *t*-test, as appropriate with GraphPad Prism v.5 software. *p-*value <0.05 was considered as statistical significance.

## For more information

Scherer Laboratory Website at UTSW Medical Center: http://www4.utsouthwestern.edu/schererlab/

## Supplementary material

As a service to our authors and readers, this journal provides supporting information supplied by the authors. Such materials are peer reviewed and may be re-organized for online delivery, but are not copy-edited or typeset. Technical support issues arising from supporting information (other than missing files) should be addressed to the authors.
