# Supplementary material for: Inhibition of endotrophin, a cleavage product of collagen VI, confers cisplatin sensitivity to tumours
Source: EMBO Mol Med. 2013 Apr 30;5(6):935–48. doi: 10.1002/emmm.201202006 (PMC3779453; doi:10.1002/emmm.201202006)
Supplement: Supplementary file 1 [file emmm0005-0935-SD1.pdf]

EMBO Molecular Medicine

Inhibition of Endotrophin, a Cleavage Product of Collagen VI, Confers Cisplatin Sensitivity to Tumors

Jiyoung Park, Thomas S. Morley and Philipp E. Scherer

Corresponding author: Philipp Scherer, The University of Texas Southwestern Medical Center

Review timeline:

Submission date:	09 September 2012
Editorial Decision:	17 November 2012
Revision received:	19 February 2013
Editorial Decision:	14 March 2013
Revision received:	17 March 2013
Accepted:	18 March 2013

Transaction Report:

(Note: With the exception of the correction of typographical or spelling errors that could be a source of ambiguity, letters and reports are not edited. The original formatting of letters and referee reports may not be reflected in this compilation.)

Editor: Anneke Funk / Roberto Buccione

1st Editorial Decision 17 November 2012

Thank you for the submission of your manuscript to EMBO Molecular Medicine. We have now heard back from the three Reviewers whom we asked to evaluate your manuscript. We are very sorry for the delay in getting back to you their comments. As I mentioned in a previous communication, in this case, unfortunately, we experienced unusual difficulties in securing three appropriate reviewers in a timely manner. Although in some exceptional cases, we can decide to proceed with two out of three evaluations, this has not proven possible in this case as the first two reports received were inconsistent.

You will see that while Reviewers 2 and 3 are generally supportive and underline the potential interest of your work, Reviewer 1 raises serious doubts on the adequacy of the model system. These, plus a number of additional concerns, prevent us from considering publication at this time.

Reviewer 1 specifically maintains that your choice of model system, the MMTV-PyMT mouse, is suboptimal to test whether endotrophin affects sensitivity to cisplatin. In addition, since cisplatin is not the standard treatment for breast cancer, s/he raises serious concerns about the overall significance of your work. Finally, Reviewer 1 is not convinced that your characterization of EMT after cisplatin treatment is fully supported by the data.

Reviewer 2 also considers that the characterization of EMT is not adequately supported by experimental evidence and feels that the data on tumour growth with combinatorial TZD and cisplatin treatment should be integrated with observations on metastasis.

Reviewer 3 is concerned that the evidence that the effects shown are truly cisplatin-specific. S/he points out that induction of EMT may increase resistance to a number of cytotoxic agents and

suggests the increase in metallothionein following cisplatin reported in supplemental Figure 1 may be indirect. S/he also questions the method used angiogenesis assessment. I concur with this Reviewer's comment that your manuscript could benefit for a more explicit narrative style that would be more accessible by a clinical readership.

Overall, it is clear that publication of the manuscript cannot be considered at this stage. Addressing the Reviewers' concerns in full will be necessary for further consideration of the manuscript in our journal and this appears to require a significant amount of additional work and experimentation. I am unsure whether you will be able or willing to address them in a revised manuscript. On the other hand, given the potential interest of the findings, I would be willing to consider a revised manuscript with the understanding that this would entail a second round of review. I should remind you that it is EMBO Molecular Medicine policy to allow a single round of revision only and that, therefore, acceptance or rejection of the manuscript will depend on the completeness of your responses included in the next, final version of the manuscript. For this reason, and to save you from any disappointament in the end I would strongly advise against returning an incomplete revision and would understand your decision if you chose to rather seek publication elsewhere at this stage.

As you know, EMBO Molecular Medicine has a "scooping protection" policy, whereby similar findings that are published by others during review or revision are not a criterion for rejection. Should you decide to submit a revised version, I do ask you to please let me know and also get in touch with us after three months if you have not completed it, to update us on the status. Please also contact us as soon as possible if similar work is published elsewhere during your revision.

***** Reviewer's comments *****

Referee #1 (Comments on Novelty/Model System):

I find the choice of model system suboptimal to address the question whether endotrophin affects cisplatin sensitivity. The MMTV-PyMT model is a quick model to induce mammary tumors, but it does not resemble any of the human breast cancer subtypes. Moreover, cisplatin is not a standard treatment of breast cancer. Hence, what do we really learn and how can we apply this info to human cancer?

Referee #1 (General Remarks):

In this manuscript the authors claim that the inhibition of endotrophin results in cisplatin sensitivity. Using the MMTV-PyMT mouse mammary tumor model, the authors investigate cisplatin responses under various conditions. Endotrophin was reduced by collagen 6 knockout, or by TZD treatment. Moreover, an endotrophin-targeting monoclonal antibody was used. In addition, the authors used transgenic mice to overexpress endotrophin in the tumor cells. Under these conditions a modulation of the cisplatin responses was observed. As mechanism, the authors claim that endotrophin induces EMT which in turn causes cisplatin resistance.

General comments

1. The model: I find the choice of model system suboptimal to address the question whether endotrophin affects cisplatin sensitivity. The MMTV-PyMT model is a quick model to induce mammary tumors, but it does not resemble any of the human breast cancer subtypes. Moreover, cisplatin is not a standard treatment of breast cancer. Hence, what do we really learn and how can we apply this info to human cancer?
2. The mechanism: I do not find the presented data convincing to state that there is an induction of EMT after cisplatin treatment (see major specific comments below).
3. I appreciate that there are differences in tumor growth after cisplatin treatment under the various conditions. However, I find the effect of endotrophin inhibition rather small. The authors do not show any clear shrinkage of established tumors. Instead, there is a delay in tumor progression or growth. Often, this delay is just a few days. Do the authors really think these minor changes justify any clinical validation of the approach? I think that there is a general overstatement of the observed

findings.

Major specific comments

1. The MMTV-PyMT mice develop several mammary tumors at the same time. How did the authors deal with this complication? Does n mean individual tumors from individual mice or are some of these tumors from the same mouse?
2. Cisplatin is mostly given when tumors are hardly palpable. The study design looks more like a chemo-preventive study. How would an established tumor of 250-500 cubic mm respond to cisplatin+endotrophin inhibition?
3. What about the effect of endotrophin on chemotherapeutic drugs that are frequently given to breast cancer patients (e.g. anthracyclins, taxanes. 5FU, cyclophosphamide)?
4. I am somewhat confused regarding the localization of endotrophin. I understand that endotrophin is generated from the ECM (a collagen cleavage product). Why do the authors then use transgenic mice in which the tumors themselves produce endotrophin? Does this have any physiological relevance?
5. IHC analyses: since the effects are small I am worried that the presented data may be biased or selected. I think the data should be analyzed blindly by 2 independent pathologists before the data are published.
6. necrosis: Fig. 4A: I agree that the areas marked by a star for the ETP/CIS condition look like necrosis. However, the other examples (CIS, TZD/CIS, and COL6-/-/CIS) look different. They look like cysts, and we do not know whether these are the result of necrosis.
7. EMT:
a. Fig. 4D: The morphology of the tumors for CIS, ETP/CIS, TZD/CIS, COL6-/-/CIS still looks like connected epithelial tissue, not mesenchymal. I am therefore surprised that there is no E-cadherin staining. Is there still E-cadherin staining after TZD treatment and in the untreated COL6-/- tumors? Unfortunately, the resolution of the IHC is poor, and I cannot assess whether the staining for E-cadherin really worked for the PBS condition.
b. Fig. 4C: in the slide for TZD/CIS there is no vimentin staining at all. This is surprising since there is clearly stroma on the slide that should be positive. For the ETP/CIS condition there are some cells that are highly positive. Are these really tumor cells and not infiltrating immune cells?
c. Fig. 4E is a model, and I do not find it appropriate to put actual bars with values that have a certain relation to each other in such a diagram. I think this part should be removed.
8. Figure 2: In A I miss the standard deviation for the Ctrl. In C I do not see a difference between COL6-/- and COL6-/-/ETP for the IVIS picture, although the bars claim a significant difference.
9. standard deviation: for all the graphical representation of tumor growth the authors use the standard error of the mean (SEM). I do not see a scientifically sound reason to use the standard error of mean for the description of data variation of repeated tumor size measurements. The reader would like to see the usual standard deviation (SD) measurement. Or does n=5 mean that 5 mice were used and for each individual mouse an average of the various tumors that were measured per mouse was taken? For the deviation of averaged values I would understand the SEM. But then the authors have to clarify how many individual tumors per mouse (n) were measured.
10. significance measurement: the group sizes are small (n is usually 5-8), and we do not know whether the data are normally distributed. Nevertheless, the authors use t test statistics to measure significance. Are the data still significant using a non-parametric test that would be more appropriate for small sized groups (e.g. Mann-Whitney)?
11. Fig. 3: there is a substantial difference between Ctrl/CIS and ETP/CIS in A (tumor progression), whereas this difference is very small in B where actually real tumors are treated. Does this not suggest that the role of endotrophin on cisplatin is more related to tumor progression? I also miss the PBS controls in Fig. B
12. Fig. 6A: What about responses of the tumors to the treatment with the anti-ETP antibody (or TZD) as single agent?

Minor comments

1. The authors usually talk about "synergistic" effects. I think that one should be careful with this term for in vivo experiments. In vitro there are defined approaches to determine synergy, but in vivo this is difficult. I would prefer if the authors addressed this as "beneficial" or something in this direction.
2. Page 5 line 11: This is still Fig. 1A, not 1B.

3. Fig. 1B: Can the Col6A3 levels also be detected in situ in the ECM of tumors?
4. For the RT-PCR analyzes only 1 reference was used (b-actin). Are the data confirmed by other references?
5. discussion, page 11, line 9: "Higher levels of endotrophin...": what is the evidence that endotrophin levels are increased in advanced breast cancer? The 3 articles cited only refer to EMT.

Referee #2 (Comments on Novelty/Model System):

This manuscript deals with a highly clinically relevant problem relating to chemoresistance of breast cancer to cisplatin, highlighting the role of the ECM in tumor progression. Precisely, the authors investigate the basis for chemoresistance using several in vivo mouse models based on the PyMT mammary tumor mouse. The main culprit shown to mediate resistance to platinum-based therapy is Endotrophin (ETP) a cleavage product of collagenVI 3 (COL6A3) which was shown by the same authors to mediate EMT in a recent JCI manuscript. Here, they show that ETP conveys cisplatin resistance. Using a variety of in vivo models based on the PyMT, they test the effect of ETP on tumor progression. These include COL6 knock-out mice and ETP overexpression to determine the adverse relationship between ETP and thiazolidinediones (TZDs) which cause downregulation of ETP. They present compelling evidence to show that all of the above manipulations including treatment with a monoclonal antibody to ETP, sensitize tumors to apoptosis by cisplatin.

Referee #2 (General Remarks):

This manuscript deals with a highly clinically relevant problem relating to chemoresistance of breast cancer to cisplatin, highlighting the role of the ECM in tumor progression. Precisely, the authors investigate the basis for chemoresistance using several in vivo mouse models based on the PyMT mammary tumor mouse. The main culprit shown to mediate resistance to platinum-based therapy is Endotrophin (ETP) a cleavage product of collagenVI 3 (COL6A3) which was shown by the same authors to mediate EMT in a recent JCI manuscript. Here, they show that ETP conveys cisplatin resistance. Using a variety of in vivo models based on the PyMT, they test the effect of ETP on tumor progression. These include COL6 knock-out mice and ETP overexpression to determine the adverse relationship between ETP and thiazolidinediones (TZDs) which cause downregulation of ETP. They present compelling evidence to show that all of the above manipulations including treatment with a monoclonal antibody to ETP, sensitize tumors to apoptosis by cisplatin.

The data are highly convincing and represent an important stepping stone into the mechanism of resistance to platinum-based therapies in breast cancer, which could be applicable to other cancers. It also point to a link between obesity and cancer as ETP is produced by the adipose tissue. There remain however a few concerns to be addressed.

1) Although they have described in a recent JCI manuscript the effects of ETP on EMT, the molecular characterization of EMT manifestation is insufficient. The authors use E-cadherin and Vimentin read outs and do not address effects on EMT transcription factors which were shown to mediate drug resistance.

2) Effects on tumor cell death was measured by histology but would benefit from showing apoptotic effects as TUNEL or caspase activity measurements.

3) The effects of combinatorial TZD and cisplatin was measured on tumor growth. What are the effects on metastasis? which might give a chance to resistant cells to escape to distant organs.

4) The manuscript would highly benefit from showing the synergy between TZDs and cisplatin in EMTing human breast cancer cell xenografts. This will add further dimension and credibility to the translational application of these findings to human breast cancer.

Referee #3 (Comments on Novelty/Model System):

No issues as preclinical model systems.

Referee #3 (General Remarks):

This is an interesting and highly clinically-relevant manuscript which provides key insights into how cisplatin therapy is potentiated by PPARgamma agonists. Following previous publications, the authors show that cisplatin therapy in a mouse model of breast cancer increases expression of COL6A3 and that response to cisplatin is modulated by COL6 as Col6-/- mice have increased sensitivity. This can be reversed by a Col6 transgene. The effects are shown to be caused by endotrophin and its effects on EMT. Detailed studies on endotrophin induced EMT are presented. Anti-endotrophin antibodies augment cisplatin sensitivity.

Specific comments

1. What is the evidence that the effects shown are truely cisplatin-specific? Induction of EMT via endotrophin may increase resistance to a wide range of cytotoxic agents and the increase in metallothionein shown in Supp Fig 1b is certainly not specific for cisplatin resistance (and may be an indirect effect). This is an important point given the title and clinical importance. Data for other cytotoxic agents should be shown.

2. No data is provided for the endotrophin transgene expression (Fig 2C). How does this compare to physiological levels?

3. The quantitation methods for the IHC and necrosis methods are not described. How comprehensive was the sampling and what was the design? Assessment of angiogenesis by counting vessels is fraught with difficulty and bias. Stereology remains the gold standard for angiogenesis endpoints. Quantitative differences in IHC assays for vimentin and other EMT markers are also hard to discern.

General comments

The manuscript is well written but is very data-dense and may not be very assessable by a clinical audience. The MS would benefit from a more explicit narrative style that highlights the implications and conclusions of previous work.

1st Revision - authors' response 19 February 2013

Referee #1 (Comments on Novelty/Model System):

I find the choice of model system suboptimal to address the question whether endotrophin affects cisplatin sensitivity. The MMTV-PyMT model is a quick model to induce mammary tumors, but it does not resemble any of the human breast cancer subtypes. Moreover, cisplatin is not a standard treatment of breast cancer. Hence, what do we really learn and how can we apply this info to human cancer?

We respectfully beg to disagree with the reviewer's assessment. The MMTV-PyMT is a widely used model in the field that has been extensively used in many high impact publications with proven relevance for the clinical breast cancer settings. As with all rodent models, it is a tool that has limitations, and we acknowledge these limitations. However, it is the one model with the most uniform progression across a cohort, both with respect to primary lesion formation as well as metastatic growth.

The Middle T antigen could be considered a mimic of a constitutively activated receptor tyrosine kinase and activates downstream PI3K, Shc and PLCγ, all of which are relevant for human breast cancer pathophysiology. In the revised version, we have performed additional xeno- and allotransplant experiments to alleviate these concerns.

With regards to cisplatin – based class of drugs not conventionally being used in the context of breast cancer, we believe that this statement is not entirely accurate either. Cisplatin and carboplatin are now used quite frequently in the treatment of some subtypes of breast cancer. Though not FDA-approved for breast cancer treatment, the platins are listed on the compendium as acceptable agents for some subtypes of breast cancers, and are in fact used for this purpose in our NCI-designated Comprehensive Cancer Center here at UTSW Medical Center.

While we reiterate these points in the specific answers below, we want to emphasize that we appreciate and very much respect to reviewer's concern and acknowledge the limitations of our model and approach, both in the answers provided here as well as in the paper. We believe though that the data presented allows us to establish a basic proof of principle for the overall approach and impact of our experimental choices and its translatability into the clinical setting.

Referee #1 (Remarks):

In this manuscript the authors claim that the inhibition of endotrophin results in cisplatin sensitivity. Using the MMTV-PyMT mouse mammary tumor model, the authors investigate cisplatin responses under various conditions. Endotrophin was reduced by collagen 6 knockout, or by TZD treatment. Moreover, an endotrophin-targeting monoclonal antibody was used. In addition, the authors used transgenic mice to overexpress endotrophin in the tumor cells. Under these conditions a modulation of the cisplatin responses was observed. As mechanism, the authors claim that endotrophin induces EMT which in turn causes cisplatin resistance.

We thank to the reviewer#1 for the critical assessment and very helpful comments towards improvement the manuscript.

General comments

1. The model: I find the choice of model system suboptimal to address the question whether endotrophin affects cisplatin sensitivity. The MMTV-PyMT model is a quick model to induce mammary tumors, but it does not resemble any of the human breast cancer subtypes. Moreover, cisplatin is not a standard treatment of breast cancer. Hence, what do we really learn and how can we apply this info to human cancer?

As outlined above, we would argue:

- **The MMTV-PyMT model system is widely used as a preclinical model of breast cancer progression, which develops tumors in distinctive stages, ranging from hyperplasia to late carcinoma, reflecting human cancer progression (Lin et al, 2003).**
- **As this reviewer points out, the MMTV-PyMT mice develop tumors aggressively. To expand tumor retention periods, we used allograft models as an alternative system.**
- **Cisplatin and carboplatin are now used quite frequently in the treatment of some subtypes of breast cancer. Though not FDA-approved for breast cancer treatment, the platins are listed on the compendium as acceptable agents for some breast cancers.**

2. The mechanism: I do not find the presented data convincing to state that there is an induction of EMT after cisplatin treatment (see major specific comments below).

- **Multiple recent reports have shown that cisplatin treatment in cancers induces EMT (Chen et al, 2011; Haslehurst et al, 2012; Latifi et al, 2011; Zhu et al, 2012). We confirm these studies and this is applicable to our current model systems as well.**
- **To more clearly indicate this, we measured the mRNA levels in tumor tissues for additional EMT markers such as snai1 (Snail), snai2 (Slug), Twist-1 and -2, E-Cadherin, N-Cadherin, and Vimentin. Particularly, mRNA levels for snail, slug, and twist1 are significantly increased in response to cisplatin treatment, and these markers are normalized with a TZD combination. These new data sets have been added to the *revised Figure 1D*.**
- **Additional immunofluorescence pictures for the EMT markers E-Cadherin and Vimentin are also included in the *revised Figure panels 1E-F*. These data strongly support the notion that cisplatin augments EMT in tumor tissues.**

3. I appreciate that there are differences in tumor growth after cisplatin treatment under the various conditions. However, I find the effect of endotrophin inhibition rather small. The authors do not show any clear shrinkage of established tumors. Instead, there is a delay in tumor progression or growth. Often, this delay is just a few days. Do the authors really think these minor changes justify any clinical validation of the approach? I think that there is a general overstatement of the observed findings.

- **As this reviewer points out, most of preclinical animal models cannot completely reflect the progression of human cancers with respect to periods of tumor incubation and development. As the MMTV-PyMT mice develop tumors very aggressively, differences of primary tumor growth among the groups occur within a range of weeks. This is the nature of the system used, and the differences observed have to be taken at face value and are statistically significant. To overcome the very aggressive nature of the MMTV-PyMT model, we have resorted to additional allograft models to allow us to monitor tumor progression in a wider window.**
- **In addition, we developed a novel tumor-imaging tool to allow us to determine tumor progression in a more sensitive approach and applied it to the model here (please see below Figure R1).**

Major specific comments

1. The MMTV-PyMT mice develop several mammary tumors at the same time. How did the authors deal with this complication? Does n mean individual tumors from individual mice or are some of these tumors from the same mouse?

We agree with the reviewer's concerns about the quantification of tumor burden in the MMTV-PyMT mice since this mouse develops multiple tumor lesions; it is correct that it is challenging to determine the tumor burden by using merely caliper measurements. We and others have addressed this issue in the following ways:

- One of the most conventional ways to determine tumor burden for the MMTV-PyMT is assessing tumor volume for inguinal tumors (#4-#5) by using caliper measurements. Thus, we also measured inguinal tumors as a standard for tumor growth. *n* reflects the numbers of individual mice used for the experiments.
- We see that the caliper measurement is not always optimal. Therefore, we generated *de novo* the MMTV-FP635 transgenic mice, expressing an infrared fluorescence protein driven by MMTV promoter, as a tumor-imaging tool. This allows us to determine tumor burden in whole body levels by measuring fluorescence signal intensity (please see below, Figure R1).
- Therefore, the original data of tumor growth that we determined with caliper measurement are replaced to representative tumor-imaging data with appropriate quantifications (Please see *revised Figures. 1A and 2A-C*).

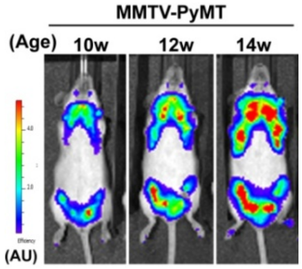

Figure R1. The MMTV-FP635 mice model as a tool for whole body tumor imaging. Longitudinal whole body *in vivo* tumor imaging with MMTV-FP635 transgenic mice (FP635/PyMT). Female MMTV-FP635 mice were crossed with male MMTV-PyMT mice to obtain female FP635/PyMT mice. Tumor volumes for the PyMT/FP635 mice at indicated time points were determined by integration of infrared fluorescence signal expressed in the ductal epithelium during tumor progression. Images were acquired with IVIS scanner. (Details are also described in our JCI paper *J. Clin Inv. (122)4243-4256, Supplementary Figure 4*).

2. Cisplatin is mostly given when tumors are hardly palpable. The study design looks more like a chemo-preventive study. How would an established tumor of 250-500 cubic mm respond to cisplatin+endotrophin inhibition?

- Cisplatin was given to 10 to 11-week-old MMTV-PyMT mice when tumors are at the early carcinoma stage as determined by histological assessment (Lin et al, 2003). Through the tumor imaging studies, tumors have clearly developed in this time point and are curable by chemotherapy (please see Figure R1, 10-week time point). The MMTV-PyMT mice in the FVB background have to be sacrificed by week 14 due to tumor burden as mandated by our Animal Welfare Protocol. We therefore wait with these experiments until the latest possible time point.
- To assess the effects of endotrophin neutralization in cisplatin regimens for tumors with a volume of 250-500 mm^3, we performed an experiment with a new cohort (please see Figure R2 below). Combining the growth inhibitory potential of endotrophin and cisplatin combined confers higher sensitivity to tumors compared to either cisplatin or 10B6 (the anti-ETP monoclonal antibody) alone.

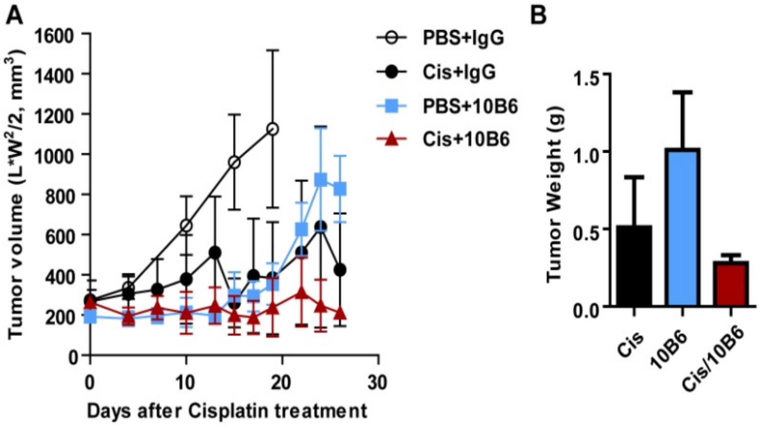

Figure R2. Tumor regression following cisplatin treatment in combination with endotrophin neutralizing monoclonal antibodies (10B6). Primary cancer cells were freshly isolated from tumors in MMTV-PyMT mice and implanted into isogenic WT mice (0.5 × 10^6 cells/mouse). Cisplatin (2mg/kg, twice a week) and either endotrophin neutralizing antibodies (10B6, 100μg/mouse/week) or IgG controls were co-administered when tumor volume reached at 200-250mm^3. A. Tumor volume is monitored two to three times a week over the tumor progression. B. Tumor weight is determined at 26 days after treatment.

Data represent mean±SD (n=5/group).

3. What about the effect of endotrophin on chemotherapeutic drugs that are frequently given to breast cancer patients (e.g. anthracyclins, taxanes. 5FU, cyclophosphamide)?

- **Mechanisms of action of chemotherapeutic drugs vary. In the studies presented here, we wanted to focus on the beneficial effects of endotrophin inhibition in the context of cisplatin treatment. This is mostly based on previous publications, including a paper that describes COL6A3 (the precursor of endotrophin) as the single most upregulated gene in the context of platinum resistance, as described in (Sherman-Baust et al, 2003; Varma et al, 2005).**

4. I am somewhat confused regarding the localization of endotrophin. I understand that endotrophin is generated from the ECM (a collagen cleavage product). Why do the authors then use transgenic mice in which the tumors themselves produce endotrophin? Does this have any physiological relevance?

- **We aim to supply endotrophin only in the mammary tumor microenvironment; hence we generated the MMTV promoter driven transgenic mice. As we appreciate, there is no mammary adipose tissue-specific transgenic promoter cassette available, so we would need to use one of our generic adipocyte-specific promoters that would drive expression in all tissues. Endotrophin is a very powerful factor that also has profound impact on adipose tissue physiology, even in the absence of a tumor. Overexpression of endotrophin in adipose tissues can cause systemic metabolic effects (this is currently a separate ongoing project in the lab). Here, we aimed to rule out systemic metabolic changes as confounding factors, and settled on ductal epithelial-specific overexpression. Since there is high-level expression of endotrophin in the tumor microenvironment, the model chosen is the best option available.**
- **Furthermore, we know that a subset of cancer cells express high levels of endotrophin.**

5. IHC analyses: since the effects are small I am worried that the presented data may be biased or selected. I think the data should be analyzed blindly by 2 independent pathologists before the data are published.

- **We appreciate the reviewer's thoughts on this. To further improve the IHC data presented in this study, we performed additional immunofluorescence stains for tumors in each of the groups (n=5/ group). We believe we present technically significantly improved staining results with a much higher resolution. We replaced the IHC data with newly obtained immunofluorescence images (see *revised Figure 4C-D and Figure 5E-F*).**
- **As recommended, the histological data assessment was performed in a blinded fashion by our clinical pathologists in the UTSW Pathology Core Facility.**

6. necrosis: Fig. 4A: I agree that the areas marked by a star for the ETP/CIS condition look like necrosis. However, the other examples (CIS,TZD/CIS, and COL6-/-/CIS) look different. They look like cysts, and we do not know whether these are the result of necrosis.

- **We have reconciled the histological analysis, including the H&E stains. Our clinical pathologists in the UTSW Pathology Core Facility defined and quantified the necrotic areas, which is represented as % of necrotic area per tumor section (n=5-6/group). The new images and quantification of the data are included in the revised *Figure 4A and Figure 5C*.**

7. EMT:
a. Fig. 4D: The morphology of the tumors for CIS, ETP/CIS, TZD/CIS, COL6-/-/CIS still looks like connected epithelial tissue, not

mesenchymal. I am therefore surprised that there is no E-cadherin staining. Is there still E-cadherin staining after TZD treatment and in the untreated COL6-/- tumors? Unfortunately, the resolution of the IHC is poor, and I cannot assess whether the staining for E-cadherin really worked for the PBS condition.

- **We re-evaluated the E-Cadherin staining for tumors in all groups by immunofluorescence (IF) staining that allows higher resolution. Now, all IHC images for the EMT studies are replaced to new IF images (please see *revised Figure 4C*).**
- **Cisplatin clearly suppresses the E-Cadherin levels, even though the cancer cells still look like the connective epithelial cells. (Please see *revised Figure 1E and 4C*). In addition, the combination of TZD with cisplatin treatment significantly restores the E-Cadherin levels (see *revised Figure 4C*).**
- **TZD or COL6$^{-/-}$ tumors without cisplatin show indeed higher levels of E-Cadherin (please see *revised Figure 1E and Figure 6C*, respectively).**

b. Fig. 4C: in the slide for TZD/CIS there is no vimentin staining at all. This is surprising since there is clearly stroma on the slide that should be positive. For the ETP/CIS condition there are some cells that are highly positive. Are these really tumor cells and not infiltrating immune cells?

- **Same as above for the Vimentin staining: We performed IF for vimentin and co-stained with Cytokeratin and DAPI (see *revised Figure 4D*). We now present data with a higher resolution, hopefully to the reviewer's satisfaction.**

c. Fig. 4E is a model, and I do not find it appropriate to put actual bars with values that have a certain relation to each other in such a diagram. I think this part should be removed.

- **We believe a model is instrumental for the reader to effectively conceptualize the bottom line of our studies. There are no "quantitative" values associated with it, merely qualitative representations. We emphasize that this is a model. We understand that philosophies differ regarding inclusions of models to outline mechanisms of action. From our perspective, it is essential to outline a minimal model that is consistent with the data presented.**

8. Figure 2: In A I miss the standard deviation for the Ctrl. In C I do not see a difference between COL6-/- and COL6-/-/ETP for the IVIS picture, although the bars claim a significant difference.

- **As we indicated above, tumor growth obtained by caliper measurements in the original Figure 2A was replaced with tumor imaging data to more clearly show the differences of tumor burden among the groups (please see *revised Figure 2A*).**
- **We had performed experiments on multiple independent cohorts for the original Figure 2C.**
  In light of the reviewer's comments, the original Figure 2C has been replaced, and we have also included the quantifications for the longitudinal progression of tumors (please see *revised Figure 2C*). Endotrophin reconstitution into COL6$^{-/-}$ clearly augments cisplatin resistance to levels comparable to control PyMT mice.
- **Pulmonary metastasis are also included in the *revised Figure 2D*.**

9. standard deviation: for all the graphical representation of tumor growth the authors use the standard error of the mean (SEM). I do not see a scientifically sound reason to use the standard error of mean for the description of data variation of repeated tumor size measurements. The reader would like to see the usual standard deviation (SD) measurement. Or does n=5 mean that 5 mice were used and for each individual mouse an average of the various tumors that were measured per mouse was taken? For the deviation of averaged values I would understand the SEM. But then the authors have to clarify how many individual tumors per mouse (n) were measured.

- **We measured a single (largest) tumor in each of the individual mice. Hence, *n* reflects the number of mice used in the study.**
- **We appreciate the reviewer's comments on the data variation. All data were reconciled and represented as suggested.**

10. significance measurement: the group sizes are small (n is usually >5-8), and we do not know whether the data are normally distributed. Nevertheless, the authors use t test statistics to measure significance. Are the data still significant using a non-parametric test that would be more appropriate for small sized groups (e.g. Mann-Whitney)?

- **A *Student t-test* was used to measure significance between groups, particularly for the quantification of immunostains and qRT-PCR data. In these cases, the number of mice was between 5-8, with a minimum of 5 data points in each individual. Therefore, group sizes are more than 25. Testing the data with *Mann-Whitney* t-test, they are still statistically significant.**
- **For *revised Figures 2A and 2D*, significance between groups are indicated by *Mann-Whitney t-test*.**

11. Fig. 3: there is a substantial difference between Ctrl/CIS and ETP/CIS in A (tumor progression), whereas this difference is very small in B where actually real tumors are treated. Does this not suggest that the role of endotrophin on cisplatin is more related to tumor progression? I also miss the PBS controls in Fig. B

- **As tumor growth of allografts (B) is slower compared to MMTV-PyMT mice (A), longer treatments need to be applied for the allografts. Long-term treatments with high doses of cisplatin (2.5 mg/ kg) caused drug toxicity. To avoid this issue, we aimed for lower concentrations of cisplatin (1 mg/kg) in the case of long-term treatment. Therefore, we used two different concentrations of cisplatin for either MMTV-PyMT mice (A) or tumor implantation (B); 2.5 mg/kg and 1 mg/kg, respectively, due to these drug toxicity issues.**

12. Fig. 6A: What about responses of the tumors to the treatment with the anti-ETP antibody (or TZD) as single agent?

- **Unfortunately, we did not include single treatment groups (either anti-ETP Ab only or TZD only) in the original experiments. However, in Figure R2, we can see a trend towards a beneficial effect of the anti-ETP antibody to cisplatin compared to anti-ETP monotherapy. Consistent with the published pre-clinical and clinical literature on TZD mono-therapy in the context of breast cancer, TZD alone does not show any beneficial effects on tumor growth arrest of regression in our systems. In contrast, in the context of the MMTV-PyMT model, it makes tumors more aggressive. We do not present that data in the paper.**
- **To directly address the requested data, we performed additional xenograft experiments with 4T-1 breast cancer cells in nude mice. The 4T-1 cell line is a metastatic and cisplatin-resistant cell type with inherently higher degrees of EMT (Holland et al, 2010; Lou et al, 2008). Tumor growth for 4T-1 cells was not affected by either cisplatin treatment alone or anti-ETP treatment alone. However, cisplatin combined with anti-ETP treatment attenuated tumor growth (*revised Figure 7B*). Prominent effects of combination treatment (cisplatin + anti-ETP) are found at the level of pulmonary metastasis (*revised Figure 7C*). Therefore, we conclude that ETP neutralization in the context of cisplatin treatment regimens for invasive cancer cells is mainly beneficial to curb metastatic growth.**

Minor comments
1. The authors usually talk about "synergistic" effects. I think that one should be careful with this term for in vivo experiments. In vitro there are defined approaches to determine synergy, but in vivo this is difficult. I would prefer if the authors addressed this as "beneficial" or something in this direction.

We modified this term to "beneficial" according to the reviewer's suggestion.

2. Page 5 line 11: This is still Fig. 1A, not 1B.

The manuscript has been corrected and adapted according to the revised figures.

3. Fig. 1B: Can the Col6A3 levels also be detected in situ in the ECM of tumors?

We have not performed any *in situ* assays in the context of this particular paper. However, we show *in situ* hybridizations that highlight primarily expression in adipocytes in our original *J. Clin Invest.* paper (*Iyengar et al., J. Clin. Invest. 2005; 115(5):1163-76*).

4. For the RT-PCR analyzes only 1 reference was used (b-actin). Are the data confirmed by other references?

The RT-PCR analysis is confirmed with both 18S and 36B4 as alternative references and trends of fold increase are unaffected by using either one of these two references. New data which are normalized with 36B4 are included in *revised Figure 1C-D*.

5. discussion, page 11, line 9: "Higher levels of endotrophin...": what is the evidence that endotrophin levels are increased in advanced breast cancer? The 3 articles cited only refer to EMT.

As the reviewer points out, the three references cited refer to EMT.

Our previous studies showed that the levels of the carboxyl-terminal epitope of Col6a3 (precursor of endotrophin) were increased in malignant breast cancers compared to benign (Iyengar et al, 2005); thus we cite this reference in this sentence.

Notably, we have extensive preliminary data on endotrophin staining in human breast cancer specimens from patients at various stages of progression and various subtypes. We have embarked on the analysis of a larger cohort with our colleagues in Surgical Oncology. So far, most mammary tumors express endotrophin at some level regardless of the tumor stage. However, fibrotic and mesenchymal-like tumors (defined phenotypically) display extraordinarily strong endotrophin signals in the tumor stroma. Also, spindle cell carcinoma of the breast expresses very high levels of endotrophin as well. This will be a separate histopathological publication, and we do not include the result in this preclinical study.

Referee #2 (Comments on Novelty/Model System):

This manuscript deals with a highly clinically relevant problem relating to chemoresistance of breast cancer to cisplatin, highlighting the role of the ECM in tumor progression. Precisely, the authors investigate the basis for chemoresistance using several in vivo mouse models based on the PyMT mammary tumor mouse. The main culprit shown to mediate resistance to platinum-based therapy is Endotrophin (ETP) a cleavage product of collagenVIα3 (COL6A3) which was shown by the same authors to mediate EMT in a recent JCI manuscript. Here, they show that ETP conveys cisplatin resistance. Using a variety of in vivo models based on the PyMT, they test the effect of ETP on tumor progression. These include COL6 knock-out mice and ETP overexpression to determine the adverse relationship between ETP and thiazolidinediones (TZDs) which cause downregulation of ETP. They present compelling evidence to show that all of the above manipulations including treatment with a monoclonal antibody to ETP, sensitize tumors to apoptosis by cisplatin.

We thank Reviewer #2 for the overall positive assessment and very helpful comments towards improvement of the manuscript

Referee #2 (Remarks):
The data are highly convincing and represent an important stepping stone into the mechanism of resistance to platinum-based therapies in breast cancer, which could be applicable to other cancers. It also point to a link between obesity and cancer as ETP is produced by the adipose tissue. There remain however a few concern to be addressed.

1) Although they have described in a recent JCI manuscript the effects of ETP on EMT, the molecular characterization of EMT manifestation is insufficient. The authors use E-cadherin and Vimentin read outs and do not address effects on EMT transcription factors, which were shown to mediate drug resistance.

- **The reviewer's comments are well appreciated. We performed qRT-PCR with tumor tissues treated with either TZD or anti-ETP antibodies in combination with cisplatin to see whether ETP inhibition attenuates the levels of transcription factors characteristic of EMT, such as Snail, Slug, twist1, and twist2 (please *revised Figure 1D and 7D*, respectively). Particularly, twist1 levels are consistently down-regulated by either TZD or anti-ETP in combination with a cisplatin regimen.**

2) Effects on tumor cell death was measured by histology but would benefit from showing apoptotic effects as TUNEL or caspase activity measurements.

- **Following the reviewer's suggestion, we determined apoptotic cell death of tumor tissues by measuring caspase-3/7 activities (please see Figure R3 below).**
- **Cisplatin augments apoptotic cell death in tumors from the MMTV-PyMT and allograft models, whereas cisplatin resistant 4T-1 cells show less of an effect. Moreover, ETP^+ tumors display enhanced anti-apoptotic activity upon cisplatin exposure compared to control-tumors. However, we believe that measuring apoptotic cell death in tumor tissues *in vivo* is much more challenging compared to *in vitro* assessments, because the cells undergoing apoptosis only transiently score positive in apoptosis assays. We have published a number of inducible pro-apoptotic systems *in vivo* in which we take advantage of forced caspase-8 dimerization in cell types such as the adipocyte, the podocyte, the cardiac myocyte of the pancreatic β cell. We refer to these mouse models as the "ATTAC" mice (Landskroner-Eiger et al, 2010; Pajvani et al, 2005; Rutkowski et al, 2013; Wang et al, 2008). Even under these conditions of highly synchronized and widespread apoptotic cell death, it is challenging to catch the cells "in the act" of undergoing apoptosis.**

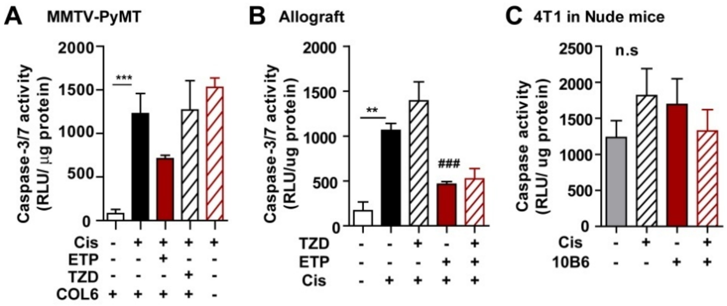

Figure R3: Apoptotic cell death in tumor tissues. Tumor tissues from the MMTV-PyMT mice (A), allografts (B), and 4T-1 xenografts (C) are homogenized and used to measure the Caspase-3/7 activities (Promega, caspase-glo 3/7 assay system). Luminescence activities are measured and normalized with protein concentration. ***$p<0.001$, ctrl vs. cis; ###$p<0.001$ ctrl+cis vs. ETP+cis by unpaired student-*t-test* (n=5~6/groups).

3) The effects of combinatorial TZD and cisplatin was measured on tumor growth. What are the effects on metastasis? which might give a chance to resistant cells to escape to distant organs.

- **This is very important comment. The most dramatic effects of TZDs on cisplatin are associated with suppression of EMT traits of cancer cells as we indicated in this study (please see *Revised Figure 1D-F*).**
- **We have determined the pulmonary metastatic growth after combination treatment with TZD and cisplatin (please see *Revised Figure 1A, metastasis*). In the MMTV-PyMT mouse model, the TZD and cisplatin combination slightly attenuates the metastatic growth.**

4) The manuscript would highly benefit from showing the synergy between TZDs and cisplatin in EMTing human breast cancer cell xenografts. This will add further dimension and credibility to the translational application of these findings to human breast cancer.

- **We prepared human breast cancer cell (MCF7) xenografts into nude mice to see if a TZD combination with cisplatin has a beneficial effect on the EMT process similar to what we have seen in mouse cancer cells. Unfortunately, all cancer cells regressed in this nude mouse cohort; we suspect that the batch of MCF7 cancer cells we used was of insufficient quality, so not even the control tumors displayed any growth. We regret that we could not include this data here. However, we are quite confident that TZDs inhibit EMT in cancer cells, as suggested by several papers (Chen et al, 2011; Haslehurst et al, 2012; Latifi et al, 2011; Zhu et al, 2012).**

Referee #3 (Comments on Novelty/Model System):
No issues as preclinical model systems.
Referee #3 (Remarks):

This is an interesting and highly clinically-relevant manuscript which provides key insights into how cisplatin therapy is potentiated by PPARgamma agonists. Following previous publications, the authors show that cisplatin therapy in a mouse model of breast cancer increases expression of COL6A3 and that response to cisplatin is modulated by COL6 as Col6-/- mice have increased sensitivity. This can be reversed by a Col6 transgene. The effects are shown to be caused by endotrophin and its effects on EMT. Detailed studies on endotrophin induced EMT are presented. Anti-endotrophin antibodies augment cisplatin sensitivity.

We thank Reviewer #3 for the overall positive assessment and very helpful comments towards improvement of the manuscript

Specific comments

1. What is the evidence that the effects shown are truly cisplatin-specific? Induction of EMT via endotrophin may increase resistance to a wide range of cytotoxic agents and the increase in metallothionein shown in Supp Fig 1b is certainly not specific for cisplatin resistance (and may be an indirect effect). This is an important point given the title and clinical importance. Data for other cytotoxic agents should be shown.

- **We appreciate the reviewer's comment. In the context of this manuscript (that already contains a very large amount of data in its present form), we focused on the effects of endotrophin on EMT in the context of cisplatin exposure. We will expand the repertoire of chemotherapeutics in separate studies.**

- **We agree with the reviewer regarding the metallothionenins, and removed the original supplementary figures on metallothioneins levels, since these are less relevant in the context of endotrophin activity on the EMT process.**

2. No data is provided for the endotrophin transgene expression (Fig 2C). How does this compare to physiological levels?

- **We determined the endotrophin transgene levels in tumors in the transgenic mice (PyMT/COL6$^{-/-}$/ETP) compared to either PyMT or PyMT/COL6$^{-/-}$ mice. No transgene was detected in PyMT or PyMT/COL6-/- mice but in PyMT/COL6$^{-/-}$/ETP (Ct values are no-detectable vs. 25-26 ranges, non-Tg mice and Tg mice, respectively).**

- **To compare the ETP transgene levels to physiological levels, we used the qRT-PCR primers detecting both endogeneous and transgene of endotrophin (primers directed against the coding region of the endogenous endotrophin). This indicates that PyMT/COL6$^{-/-}$/ETP mice express about 5-fold higher levels of ETP compared to PyMT control (*Figure R4* below). PyMT/COL6$^{-/-}$ mice express 3-fold higher Col6a3 compared to the PyMT control, which may be due to a compensatory increase. We included the qRT-PCR data in the *Revised Figure 2B*.**

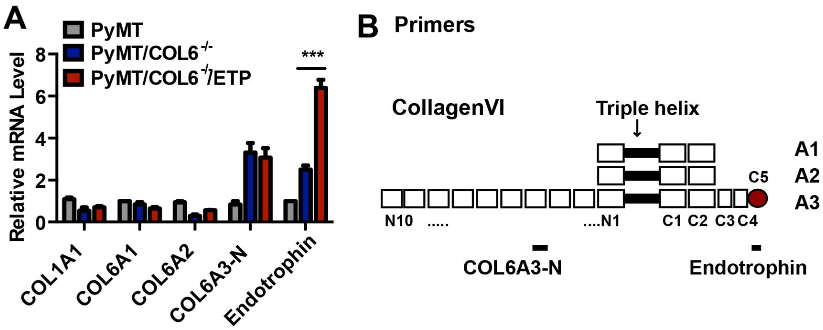

Figure R4: Endotrophin transgene levels. (A) mRNA levels of endotrophin are determined by qRT-PCR in tumor tissues. Tumor tissues of PyMT/COL6$^{-/-}$/ETP mice specifically overexpress about 5-fold higher levels of endotrophin, but not the rest of the Col6a3 chain (COL6A3-N). Results are normalized with 36B4 and represented as mean±SD. ***p<0.001, either PyMT or PyMT/COL6$^{-/-}$ vs. PyMT/COL6$^{-/-}$/ETP by unpaired student *t-test*. N=4/groups. (B) Diagram for the primers used in this figure (black boxes).

3. The quantitation methods for the IHC and necrosis methods are not described. How comprehensive was the sampling and what was the design? Assessment of angiogenesis by counting vessels is fraught with difficulty and bias. Stereology remains the gold standard for angiogenesis endpoints. Quantitative differences in IHC assays for vimentin and other EMT markers are also hard to discern.

- **We reconcile the IHC analysis with our pathologists in the UTSW pathology core facility. Necrotic areas in tumor tissues are defined by pathologists blinded to the origin of the H&E stains and are quantified in a blinded fashion. Revised images and quantified data are now included (please see *revised Figure 4A and 5C*).**

- **IHC staining of EMT markers such as E-cadherin and Vimentin are replaced with immunofluorescence images to show a higher resolution and quantified data accordingly (please see *Revised Figure 1E-F, Figure 4C-D, and Figure 5D*).**

- **Angiogenesis is determined by functional vessel density by using lectin perfusion. Confocal images are represented in *revised Figure 5E*.**

General comments
The manuscript is well written but is very data-dense and may not be very assessable by a clinical audience. The MS would benefit from a more explicit narrative style that highlights the implications and conclusions of previous work.

We have modified the manuscript thoroughly according to the reviewer's comments and have made it hopefully more palatable to the readers.

References used

Chen X, Lingala S, Khoobyari S, Nolta J, Zern MA, Wu J (2011) Epithelial mesenchymal transition and hedgehog signaling activation are associated with chemoresistance and invasion of hepatoma subpopulations. *J Hepatol* **55:** 838-845

Haslehurst AM, Koti M, Dharsee M, Nuin P, Evans K, Geraci J, Childs T, Chen J, Li J, Weberpals J, Davey S, Squire J, Park PC, Feilotter H (2012) EMT transcription factors snail and slug directly contribute to cisplatin resistance in ovarian cancer. *BMC Cancer* **12:** 91

Holland SJ, Pan A, Franci C, Hu Y, Chang B, Li W, Duan M, Torneros A, Yu J, Heckrodt TJ, Zhang J, Ding P, Apatira A, Chua J, Brandt R, Pine P, Goff D, Singh R, Payan DG, Hitoshi Y (2010) R428, a selective small molecule inhibitor of Axl kinase, blocks tumor spread and prolongs survival in models of metastatic breast cancer. *Cancer Res* **70:** 1544-1554

Iyengar P, Espina V, Williams TW, Lin Y, Berry D, Jelicks LA, Lee H, Temple K, Graves R, Pollard J, Chopra N, Russell RG, Sasisekharan R, Trock BJ, Lippman M, Calvert VS, Petricoin EF, 3rd, Liotta L, Dadachova E, Pestell RG, Lisanti MP, Bonaldo P, Scherer PE (2005) Adipocyte-derived collagen VI affects early mammary tumor progression in vivo, demonstrating a critical interaction in the tumor/stroma microenvironment. *J Clin Invest* **115:** 1163-1176

Landskroner-Eiger S, Park J, Israel D, Pollard JW, Scherer PE (2010) Morphogenesis of the developing mammary gland: stage-dependent impact of adipocytes. *Developmental biology* **344:** 968-978

Latifi A, Abubaker K, Castrechini N, Ward AC, Liongue C, Dobill F, Kumar J, Thompson EW, Quinn MA, Findlay JK, Ahmed N (2011) Cisplatin treatment of primary and metastatic epithelial ovarian carcinomas generates residual cells with mesenchymal stem cell-like profile. *J Cell Biochem* **112:** 2850-2864

Lin EY, Jones JG, Li P, Zhu L, Whitney KD, Muller WJ, Pollard JW (2003) Progression to malignancy in the polyoma middle T oncoprotein mouse breast cancer model provides a reliable model for human diseases. *Am J Pathol* **163:** 2113-2126

Lou Y, Preobrazhenska O, auf dem Keller U, Sutcliffe M, Barclay L, McDonald PC, Roskelley C, Overall CM, Dedhar S (2008) Epithelial-mesenchymal transition (EMT) is not sufficient for spontaneous murine breast cancer metastasis. *Developmental dynamics : an official publication of the American Association of Anatomists* **237:** 2755-2768

Pajvani UB, Trujillo ME, Combs TP, Iyengar P, Jelicks L, Roth KA, Kitsis RN, Scherer PE (2005) Fat apoptosis through targeted activation of caspase 8: a new mouse model of inducible and reversible lipoatrophy. *Nat Med* **11:** 797-803

Rutkowski JM, Wang ZV, Park AS, Zhang J, Zhang D, Hu MC, Moe OW, Susztak K, Scherer PE (2013) Adiponectin promotes functional recovery after podocyte ablation. *Journal of the American Society of Nephrology : JASN* **24:** 268-282

Sherman-Baust CA, Weeraratna AT, Rangel LB, Pizer ES, Cho KR, Schwartz DR, Shock T, Morin PJ (2003) Remodeling of the extracellular matrix through overexpression of collagen VI contributes to cisplatin resistance in ovarian cancer cells. *Cancer Cell* **3:** 377-386

Varma RR, Hector SM, Clark K, Greco WR, Hawthorn L, Pendyala L (2005) Gene expression profiling of a clonal isolate of oxaliplatin-resistant ovarian carcinoma cell line A2780/C10. *Oncol Rep* **14:** 925-932

Wang ZV, Mu J, Schraw TD, Gautron L, Elmquist JK, Zhang BB, Brownlee M, Scherer PE (2008) PANIC-ATTAC: a mouse model for inducible and reversible beta-cell ablation. *Diabetes* **57:** 2137-2148

Zhu K, Chen L, Han X, Wang J (2012) Short hairpin RNA targeting Twist1 suppresses cell proliferation and improves chemosensitivity to cisplatin in HeLa human cervical cancer cells. *Oncol Rep* **27:** 1027-1034

2nd Editorial Decision 14 March 2013

Thank you for the submission of your revised manuscript to EMBO Molecular Medicine. We have now received the enclosed reports from the Reviewers that were asked to re-assess it. As you will see the reviewers are now supportive and I am pleased to inform you that we will be able to accept your manuscript pending the following final amendments:

1) The description of all reported data that includes statistical testing must state the actual P value for each test (not merely 'significant' or 'P < 0.05').

2) There is space at the end of each article to list relevant web links for further consultation by our readers ("For more information"). Could you identify some relevant ones and provide such information as well? Some examples are patient associations, relevant databases, OMIM/proteins/genes links, author's websites, etc... (this is not compulsory and might not apply in your case)

Please submit your revised manuscript within two weeks. Needless to say, the sooner we receive it the sooner I will be able to formally accept your manuscript; ideally, if I receive the amended manuscript by today/tomorrow morning and provided the changes are correct, I will be able to accept within tomorrow.

***** Reviewer's comments *****

Referee #1 (General Remarks):

I am satisfied by the responses to my comments.

Referee #2 (General Remarks):

The authors have made substantial improvements on the manuscript and have clarified and responded to most reviewers comments. It is my opinion that this manuscript is in a acceptable form for publication.

Referee #3 (General Remarks):

No further comments.

2nd Revision - authors' response 17 March 2013

In response to your suggestions, we have:

A) Stated the actual P values for each test perform for statistical analysis.

B) Added a "For more information" section at the end.

We hope that with these minor modifications, the manuscript is deemed fully acceptable.
