# Supplementary material for: Inhibition of endotrophin, a cleavage product of collagen VI, confers cisplatin sensitivity to tumours
Source: EMBO Mol Med. 2013 Apr 30;5(6):935–48. doi: 10.1002/emmm.201202006 (PMC3779453; doi:10.1002/emmm.201202006)
Supplement: Supplementary file 2 [file emmm0005-0935-SD2.pdf]

Supporting Information

Inhibition of Endotrophin, a Cleavage Product of Collagen VI, Confers Cisplatin Sensitivity to Tumors

Jiyoung Park[1], Thomas S. Morley[1] and Philipp E. Scherer[1, 2, 3*]
[1]Touchstone Diabetes Center, Departments of Internal Medicine and [2]Cell Biology and [3]Simmons Cancer Center, The University of Texas Southwestern Medical Center, Dallas, Texas 75390, USA.

Table of content

1. Figure S1. TZD confers beneficial effects on cisplatin treatment in mammary tumors
2. Figure S2. The absence of COL6 sensitizes tumors to cisplatin treatment
3. Table S1. Primer sequences used for qRT-PCR

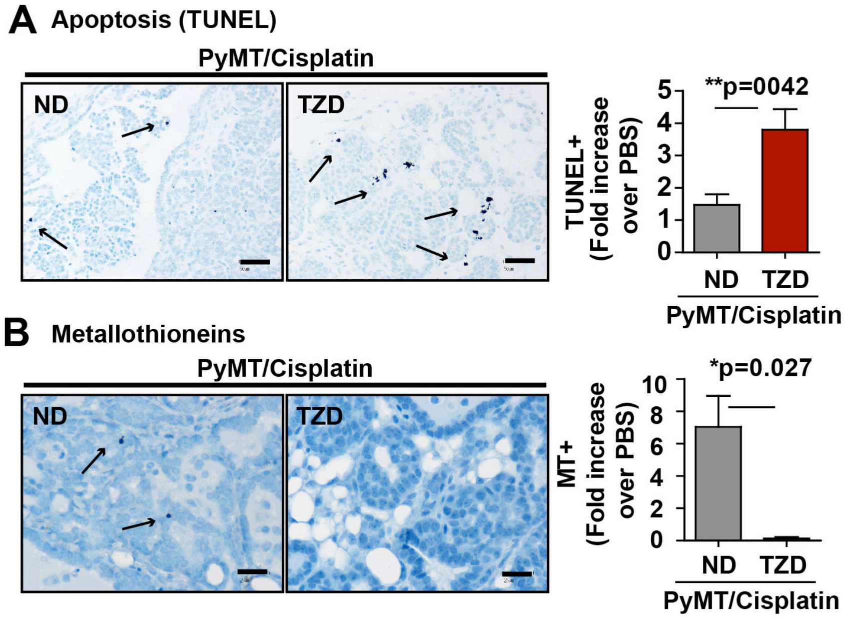

Fig. S1. TZD confers beneficial effects on cisplatin treatment in mammary tumors. Met-1 cancer cells (0.5×10^6 cells/ mouse) were implanted into wild-type mice and TZD (20 mg/ kg) was given by diet and cisplatin (1 mg/ kg) was intraperitoneally (ip.) injected every 5 days from 30 days post implantation (n=8/ group). ***p<0.001 vs. ND by *2-way ANOVA*. Tumor tissues from PyMT mice given TZD or normal diet (ND) diet were histologically assessed. Representative images and quantification for TUNEL staining (**A**), showing increased cancer cell apoptosis by TZD combination in cisplatin. Data represent mean ± SD (n=5/ group). **p=0.0042 ND vs. TZD by *unpaired student t-test*. Scales: 50 μm. Representative metallothioneins staining and quantification (**C**), showing a higher signal in tumor tissues following cisplatin, which is efficiently suppressed by TZD combination. Data represent mean ± SD (n=5/ group). *p=0.002 ND vs. TZD by *unpaired student t-test*. Arrows indicate staining positive cells. Scales: 25 μm.

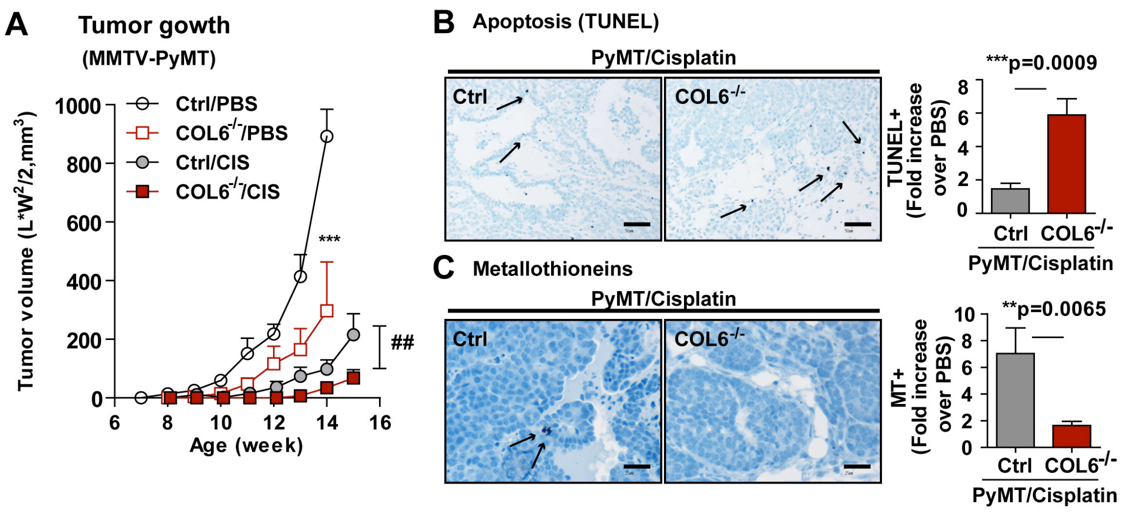

Fig. S2. The absence of COL6 sensitizes tumors to cisplatin treatment. A. 10-weeks aged PyMT and PyMT/COL6$^{-/-}$ mice were given cisplatin (1 mg/kg, ip., 2 times/ week) over tumor progression. Tumor growth was determined by caliper measurements. Data represent mean ± SD (n=8/ group). ***p<0.001 Ctrl/PBS vs. COL6$^{-/-}$/PBS; $^{\#\#}$p<0.01 Ctrl/CIS vs. COL6$^{-/-}$/CIS by *2-way ANOVA*. **B**. Representative TUNEL staining images and quantification, showing significantly increased cancer cell apoptosis in PyMT/COL6$^{-/-}$ mice by cisplatin treatment. Data represent mean ± SD (multiple images from n=5/ group). ***p<0.0009 Ctrl vs. COL6$^{-/-}$ by *unpaired student t-test*. Scales: 50 μm. **C**. Representative metallothioneins staining and quantification, showing a higher signal in tumor tissues following cisplatin which is efficiently suppressed in COL6$^{-/-}$ mice. Data represent mean ± SD (n=5/ group). **p<0.0065 Ctrl vs. COL6$^{-/-}$ by *unpaired student t-test*. Arrows indicate staining positive cells. Scales: 25 μm.

Table S1. Primer sequences used for qRT-PCR

Gene	Sense	Antisense
Snail (Snai1)	CCCTTCAGGCCACCTTCTTTGAGGT	GTCCAGTAACCACCCTGCTG
Slug (Snai2)	CTGTATGGACATCGTCGGCAG	ACTTACACGCCCCAAGGATG
Twist1	CGGCCAGGTACATCGACTTC	TGCAGCTTGCCATCTTGGAG
Twist2	TCAGCAAGATCCAGACGCTC	CTGAGATGTGCAGGTGGGTC
E-Cadherin (Cdh1)	CGATTACGAGGGCAGTGGTT	AGTCCCCTAGTCGTCCTCAC
N-Cadherin (Cdh2)	GGCAATCCCACTTATGGCCT	TCCGTGACAGTTAGGTTGGC
Vimentin	GCCAGCAGTATGAAAGCGTG	ACCTGTCTCCGGTACTCGTT
S100A4	TTGTGTCCACCTTCCACAAA	TGTTGCTGTCCAAGTTGCTC
COL6A3-N	ACGCCCATCACCACTCTAAC	CTAAACTGCACGACCCCAAT
36B4	GGCATGCGGCCCGTCTCTC	CTTCCCTGGGCATCACGGCG
